# Low-Cost Particulate Matter Sensors for Monitoring Residential Wood Burning

**DOI:** 10.1021/acs.est.3c03661

**Published:** 2023-09-27

**Authors:** Amirhossein Hassani, Philipp Schneider, Matthias Vogt, Núria Castell

**Affiliations:** The Climate and Environmental Research Institute NILU, P.O. Box 100, Kjeller 2027, Norway

**Keywords:** air pollution, uEMEP air quality model, PM_2.5_ spatiotemporal variation, data assimilation, gravimetric method, Airly sensor, calibration

## Abstract

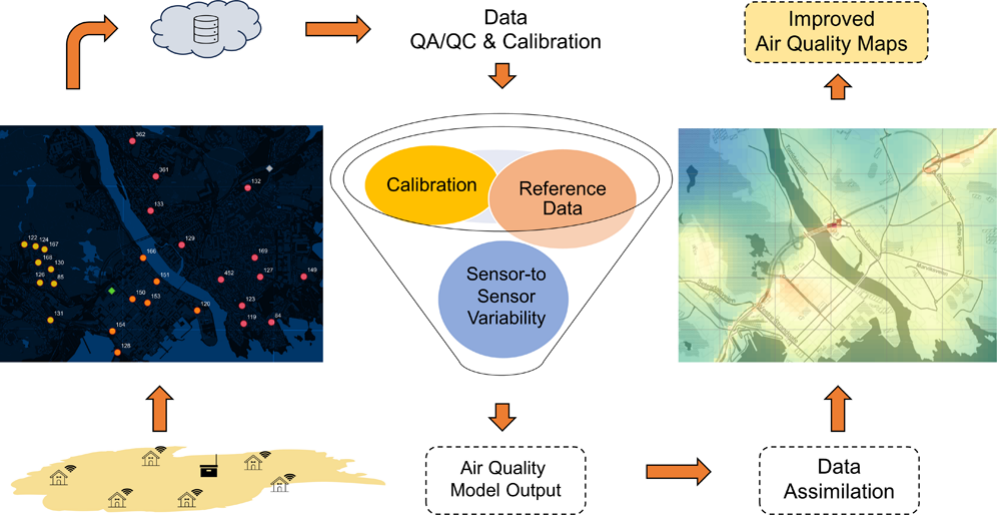

Conventional monitoring systems for air quality, such as reference stations, provide reliable pollution data in urban settings but only at relatively low spatial density. This study explores the potential of low-cost sensor systems (LCSs) deployed at homes of residents to enhance the monitoring of urban air pollution caused by residential wood burning. We established a network of 28 Airly (Airly-GSM-1, SP. Z o.o., Poland) LCSs in Kristiansand, Norway, over two winters (2021–2022). To assess performance, a gravimetric Kleinfiltergerät measured the fine particle mass concentration (PM_2.5_) in the garden of one participant’s house for 4 weeks. Results showed a sensor-to-reference correlation equal to 0.86 for raw PM_2.5_ measurements at daily resolution (bias/RMSE: 9.45/11.65 μg m^–3^). High-resolution air quality maps at a 100 m resolution were produced by combining the output of an air quality model (uEMEP) using data assimilation techniques with the network data that were corrected and calibrated by using a proposed five-step network data processing scheme. Leave-one-out cross-validation demonstrated that data assimilation reduced the model’s RMSE, MAE, and bias by 44–56, 38–48, and 41–52%, respectively.

## Abstract 2

Residential wood/biomass burning is often poorly captured by the national ambient air quality monitoring networks. Results here show that citizen-operated novel environmental technologies can contribute to monitoring in situ wood combustion air pollution in urban settings.

## 1 Introduction

Residential wood combustion/burning (RWC) in stoves, small boilers, and fireplaces are widely used for heating and for creating a cozy atmosphere in residences in the continental Nordic countries: Denmark, Finland, Norway, and Sweden;^1^ it is also common in other regions in Europe^2^ and worldwide.^3^ Due to the incomplete combustion conditions and lack of emission control devices, RWC is also a critical emission source of air pollution, mainly fine particulate matter (PM),^4^ which contains substances such as polycyclic aromatic hydrocarbons (PAHs) known to be linked to adverse health effects.^5^ Sigsgaard, Forsberg, Annesi-Maesano, Blomberg, Bølling, Boman, Bønløkke, Brauer, Bruce, and Héroux et al.^6^ present evidence that emissions from biomass, including residential solid fuels (RSFs), such as wood crop residue, animal waste, coal, and charcoal, and combustion products negatively affect respiratory and, possibly, cardiovascular health in Europe. The current impact of biomass smoke, primarily from wood, on Europe’s premature mortality is estimated at least 40,000 deaths per year.^6^ Given this context, it is concerning that RWC is increasingly perceived as a clean and inexpensive fuel when sourced locally in response to climate change policies, fuel pricing, and poverty, thus posing new health challenges.

Traditional methods for evaluating regional air pollutant trends, such as regulatory reference instruments, struggle to effectively account for the spatial and temporal variations of RWC. This is due to the large number of households using RWC appliances and the lack of precise information on appliance locations, stove technologies, and usage patterns. Additionally, the limited coverage of reference stations and the reliance of air quality models on accurate input data, meteorological conditions, boundary conditions, and emissions further hinder their ability to capture the extensive areas affected by RWC pollution.

The latest developments in low-cost air pollution technologies can offer further insight into nearby sources, assist in placing regulatory monitoring stations, and enhance our understanding of the finer-scale spatiotemporal fluctuations of ambient air pollutants and their corresponding health impacts.^7^ The cost-effectiveness of low-cost air quality sensors (LCSs) has led to an easier way of collecting data at higher spatial and temporal resolutions (throughout this paper, by a sensor, we mean a sensor system or sensor kit since they usually include a collection of sensors). LCSs have been used to supplement ambient air monitoring^8^ and improve the understanding of air quality and health in urban areas.^9^

Here, we assess the use of Airly LCS technology against reference gravimetric Kleinfiltergerät methods (KFG) for analyzing the spatiotemporal variation of PM in Kristiansand, Norway, during winter. Previous studies conducted by Im, Christensen, Nielsen, Sand, Makkonen, Geels, Anderson, Kukkonen, Lopez-Aparicio, and Brandt,^10^ Grythe, Lopez-Aparicio, Vogt, Vo Thanh, Hak, Halse, Hamer, and Sousa Santos,^11^ Kukkonen, López-Aparicio, Segersson, Geels, Kangas, Kauhaniemi, Maragkidou, Jensen, Assmuth, and Karppinen,^12^ and Lopez-Aparicio and Grythe^13^ have indicated that RWC accounts for 50–80% of house heating PM_2.5_ emissions in the Nordic area (except for Iceland). Additionally, in circumpolar regions, the RWC stands out as a prominent contributor to winter carbonaceous aerosol emissions.^14^ This study focuses on air pollution caused by RWC, an important environmental issue, and the use of PM LCSs at citizens’ houses to complement official monitoring stations and generate high-resolution air quality maps. While the use of PM LCSs for monitoring air quality is not new, the use of a commercial LCS, i.e., the Airly sensor system, has not been extensively studied in this context.

In addition to Kristiansand as the primary research location, data from additional sites/air quality stations in Oslo, Gothenburg, and Lappeenranta were utilized to evaluate Airly LCSs. The research in the areas mentioned above is part of the NordicPATH project—Nordic Participatory, Healthy, and People-Centered Cities (https://nordicpath.nilu.no/, accessed May 2023). The paper also proposes a data processing scheme for network data quality assurance. It assimilates the PM_2.5_ data from the sensor network with the uEMEP model (see Supporting Information SI.1 for uEMEP details), providing better estimates of the spatial variation of air pollution at regional scales.

## 2 Methods

### 2.1 Airly LCS and Sensor Network Description

The Airly-GSM-1 sensor system (or sensor kit/unit) (https://airly.org/en/, accessed in Nov 2022) was selected for this research. This sensor kit is a commercial platform that provides estimates of PM mass concentrations in the fractions of PM_1_, PM_2.5_, and PM_10_ as output. The Airly PM LCS integrates the Plantower PMS5003 sensor (https://www.plantower.com/en/products_33/74.html, accessed in Nov 2022) with a scattering angle of 90° (see Supporting Information SI.2 for further details).

**Figure 1:**
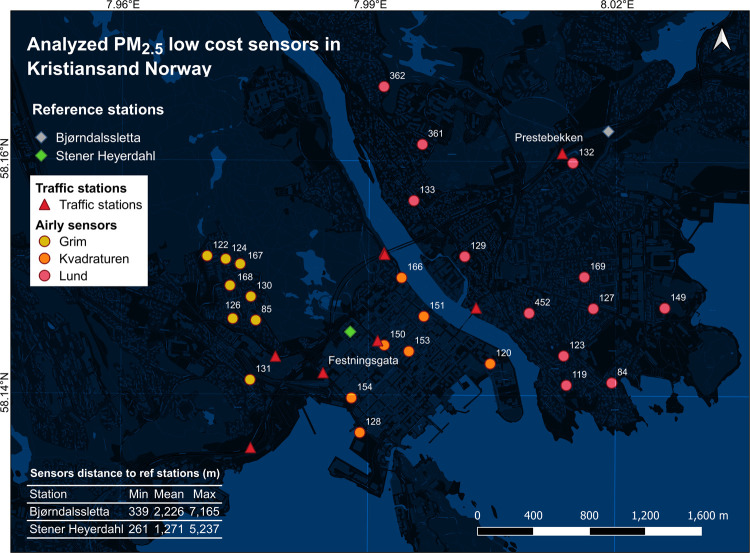
Location of Airly low-cost particulate matter sensors, reference monitoring stations, and inductive loop traffic counters, Kristiansand, Norway. Different colors for low-cost sensors represent the district (neighborhood) in which each sensor is located.

The Airly sensor network in Kristiansand has 30 Airly LCSs, with 28 of them located in the central area of the municipality, encompassing three main regions: Grim, Lund, and Kvadraturen (Figure 1). The sensor network was fully operational from Dec 2020 (17 LCSs by 11th Dec 2020). However, some sensors were added a posteriori: 5 LCSs in Jan 2021, 4 LCSs in Feb 2021, 3 sensors in June 2021, and one sensor in Jan 2022 (Supporting Table 1). Data coverage by individual LCSs is represented in Supporting Figure 1.

Sensors were installed near local RWC sources in residential areas with less traffic, typically where the RWC for heating is more prevalent. Due to proximity to the emission sources, the PM_2.5_ levels measured by LCSs during winters are believed to be dominated by RWC PM_2.5_, which contributes to ≈67.9% of total annual emissions of PM_2.5_ in Kristiansand municipality (https://www.miljodirektoratet.no/globalassets/publikasjoner/m1494/m1494.pdf).

In Kristiansand, RWC mainly occurs in detached and semidetached households, more present in the regions of Lund and Grim. However, some block buildings in the center (i.e., Kvadraturen) might also have wood stoves. We hypothesized that Grim would have high levels of air pollution in winter due to the type of households (detached) and the neighborhood’s topography, where the houses are located in a small valley and episodes with thermal inversions might reduce pollution dispersion. Following that hypothesis, we decided to have a dense network of LCSs in Grim with eight sensors. The neighborhood of Lund, especially the southern region, is more open, and dispersion might be favored by the presence of sea wind, resulting in lower air pollution levels.^15^

Kristiansand municipality has two reference air quality stations monitoring PM_2.5_ and PM_10_ (Figure 1): (1) Bjørndalssletta (traffic station) and (2) Stener Heyerdahl (background station). These stations are instrumented with Grimm EDM180 optical dust monitor devices, measuring PM_2.5_ and PM_10_ at an hourly resolution. The average distance of the LCSs to Bjørndalssletta and Stener Heyerdahl reference stations was 2226 and 1271 m, respectively.

### 2.2 Data Uncertainty against the Reference Method

To evaluate the sensor kits, we deployed the gravimetrical PM reference method—Kleinfiltergerät LVS3 and MVS6 (KFG, https://www.leckel.de/; see Supporting Information SI.3 for further details) in the backyard of one of the households in Kristiansand that also had an Airly LCS installed (ID 124). The household belongs to the Grim neighborhood, where eight sensor kits were deployed a short distance from each other (with an average sensor-to-sensor distance of 391.19 m).

The KFG device collected PM_2.5_ mass concentration data during two periods of two consecutive weeks in 2021-01-21–2021-02-03 and 2021-02-17–2021-03-02. The daily averaged PM_2.5_ mass concentrations of 28 days were compared to the sensor data from the sensor installed at the same household (ID 124) and with the average of the five LCSs (IDs: 85, 122, 124, 126, and 130) installed at the time in the vicinity to assess the accuracy of the sensor network.

We employed widely used statistical measures, including the coefficient of determination (; *y*_*i*_ is the reference value,  is the sensor measurement, and  is the mean reference value), mean absolute error (, *n* is the number of observations), root-mean-square error (), and mean bias () to evaluate the performance of the LCSs against the reference KFG instrument.

### 2.3 Network Data Quality Assurance

We retrieved data from 28 LCSs (Figure 1) from 17 Nov 2020 until 04 Sep 2022 from all three neighborhoods of Kristiansand. Sensor data coverage during that period is represented in Supporting Figure 1. We proposed a five-step scheme of data processing to ensure network data quality. In contrast to the data screening methodologies proposed by Kelly, Xing, Sayahi, Mitchell, Becnel, Gaillardon, Meyer, and Whitaker^16^ or Lu, Giuliano, and Habre,^17^ our data processing scheme offers the advantage of being applicable even in situations where there are no two sensors available per node. In the first step, (1) we removed the unwanted data from relocated LCSs (here, LCS IDs 125 and 126). These sensors were relocated during the analysis period, and postrelocated data were irrelevant to our region of interest. In the next step, (2) if the data coverage of a sensor for a specific month was less than 365 h (namely, 50% of the month), we removed that sensor’s whole month’s data. The size of data records was reduced from 342,947 to 337,179 (1.68%) during this step. In the third step, (3) we calculated Pearson’s linear correlation (*r*) between each sensor’s hourly PM measurements and the hourly average of all sensors’ measurements within a month during the nighttime hours (00:00–4:59); if the computed *r* was ≤0.7, we removed the data of the sensor during that month. We lost 12.89% of sensor data during this step, as well.

The choice of nighttime hours is based on the fact that fewer anthropogenic activities may occur that can contribute to fluctuations in PM_2.5_ concentrations. For example, less RWC may happen, traffic may be lighter during the night hours, and industrial activities may be reduced. As a result, PM_2.5_ concentrations during nighttime hours may be more similar to the background levels. Supporting Figure 2 represents the diurnal *r* between Stener Heyerdahl and Bjørndalssletta. It is based on historical PM_2.5_ data starting in 2020. During night hours between 00:00 and 4:59, we observe the highest *r* between the measurements from the two reference stations. This indicates that during these hours fewer activities contribute to local PM_2.5_ emissions, resulting in a higher spatial consistency and similarity in observed PM_2.5_ to background values.

Other studies analyzing environmental sensor network data have adopted the practice of examining the *r* between individual sensors and the sensor network. For example, Fu, Tang, Grieneisen, Yang, Yang, Wu, Wang, and Zhan^18^ removed the sites with substantially lower *r*s than any other site, where the threshold of the *r* was set to be lower than μ-3σ (μ: mean, σ: standard deviation) of all of the *r*s between sites. In our case, the mean and standard deviation of the *r*s of all sensors during the analysis period (2020-12-01 00:00:00–2022-08-31 23:00:00) were 0.74 and 0.02, respectively. Additionally, in urban settings, PM_2.5_ concentrations have been shown by some studies to be spatially correlated.^19^ Using *r*s between sites is a popular method of determining spatial uniformity in urban areas.^20^ A summary of more studies on PM_2.5_ spatial correlation (using reference measurements) is provided in Supporting Table 2. While there may be a high correlation between concentrations at pairs of sites, it is important to emphasize that their actual concentrations are not necessarily identical.

A survey on sensor calibration in air pollution monitoring deployments by Maag, Zhou, and Thiele^21^ categorizes this approach as the “Blind Network Calibration” approach (see Table 2 in their paper for the list of studies that used blind calibration, tailored explicitly for air quality sensors). It is assumed that neighboring sensors measure almost identical values or at least are correlated with each other^21,22^ as environmental data collected from widely dispersed sensors have similar temporal and spatial characteristics.^23,24^

Through a series of 7 week sensor-to-sensor intercomparison tests for Airly LCSs, Vogt, Schneider, Castell, and Hamer^25^ showed that different Airly LCSs are well correlated among them, with *r*s between 0.89 and 0.96. To evaluate the consistency in measurements of Airly LCSs, we conducted a sensor-to-sensor intercomparison test in Oslo. The intercomparison consisted of deploying 16 sensors at a single location, and it allowed us to evaluate the consistency and *r* between different sensors’ measurements. Supporting Figure 3 illustrates the findings of the intercomparison, showcasing the *r* between the sensor measurements in terms of measuring PM_2.5_. The intercomparison test is conducted in Kirkeveien, Oslo, with meteoclimatic conditions similar to Kristiansand for nearly 20 days (2023-04-12 until 2023-05-01). However, those are not the sensors used in Kristiansand. Fifteen sensors showed a sensor-to-sensor *r* above 0.99, except sensor ID 528, which showed a relatively lower performance; even for that sensor, sensor-to-sensor *r*s were above 0.78.

Based on the above sensor-to-sensor variability—although the sensors in Kristiansand were from a different batch, and the comparison results against the KFG instrument (discussed in detail in Section 3)—we corrected all of the Airly sensors’ PM_2.5_ measurements in Kristiansand in the fourth step by multiplying the sensor factory-calibrated outputs by 0.49 as a correction factor (the sensor gain calculated from sensor ID 124 colocated with the KFG). Then, Stener Heyerdahl (background station) was used as a reference for adjusting the weekly bias (offset) of the sensor PM_2.5_ measurements.

In the fifth step, an assessment was conducted to determine the potential sensor drift. Following the filtering and calibration steps, we used singular spectrum analysis (SSA)^26^ to identify the long-term trend, the seasonal or oscillatory trend (or trends), and the remainder of each sensor nightly bias (00:00–04:59) from the urban background Stener Heyerdahl reference monitoring station from the beginning of the first winter to the end of the second winter. This was performed only for the sensors with at least 90 days of data in a row to establish an LCS baseline (26 sensors). We fitted a linear regression model to the measurements of individual sensors and assessed the slope coefficient’s *p*-value. If it was >0.05, we considered the sensor as nondrifted. For none of the sensors was the slope coefficient of the fitted line statistically significant.

### 2.4 Data Assimilation Methods for Air Quality Mapping

One promising method to exploit the observations from networks of LCSs is to combine their measurements with the information provided by a high-resolution air quality model. Unless the sensor network is very dense, a model is required to realistically interpolate between the observations. At the same time, the model benefits from the correction of potential biases with actual measurements.

Over time, various methods have been developed for this task, including data fusion methods based on geostatistics^27^ and data assimilation.^28^ Here, we use the optimal interpolation (OI) approach, one of the most basic data assimilation techniques and has seen widespread adoption and use in numerical weather prediction for several decades. First proposed by Gandin,^29^ OI involves merging two data sets, a priori field (usually from a model) and observations, by applying objective weights based on their respective uncertainties to create an analysis field (see Supporting Information SI.4 for further details on OI).

## 3 Results and Discussion

### 3.1 Comparison of LCS Output and Reference (KFG)/Reference-Equivalent Methods

Supporting Figure 5 shows the calibration results for sensor ID 124, including and excluding the intercept and relative humidity (RH) in linear calibration equations. The RH coefficients and intercept are statistically insignificant according to the *p*-values (*p* < 0.05). Accordingly, in the calibration of the sensors (step 4 in the data processing scheme), we did not include RH and intercept. This might be related to the fact that we use 24 h averages.

Unfortunately, the Airly PM LCSs were not colocated at the reference monitoring stations in Kristiansand during the winter. To evaluate the efficiency of the proposed data processing scheme, we collected data from other colocated sensors in the traffic Haga site in Gothenburg, Sweden (Lon: 11.96054, Lat: 57.69785), and urban background Pekkasenkatu station in Lappeenranta (Lon: 28.24585, Lat: 61.05748), Finland, from the same Airly sensor batch. Having a close distance (200 km) to Kristiansand, Gothenburg has a climate similar to Kristiansand. The results of colocation at the Haga station and data correction are represented in Supporting Figure 6 (in all following figures, the sensor-ref indicates the raw output of the sensor compared to the reference measurements). We first corrected the sensor data in the Haga station by multiplying the raw sensor output by 0.49 and then corrected the mean weekly bias from the reference measurements. We computed the means of both sensor and reference measurements, and the mean of the sensor measurements was shifted to match the reference mean. Our correction approach reduced the hourly sensor-ref MAE from 4.6 to 2.17 μg m^–3^ and the hourly sensor-ref RMSE from 7.02 to 2.87 μg m^–3^.

The uncertainty introduced by RH to the output of Airly LCSs colocated at Haga and Pekkasenkatu stations before and after the 5-step data processing scheme is represented in Supporting Figure 7. We fitted normal distributions to the sensor biases before and after correction at different RH bins—60:10:100%. For example, for the sensor colocated at the Haga station, the applied correction reduces the MB from 4.34 μg m^–3^ (95% confidence intervals: 3.97, 4.82) to 0.23 μg m^–3^ (0.11, 0.33) at the RH bin of 90–100%. Hofman, Peters, Stroobants, Elst, Baeyens, Van Laer, Spruyt, Van Essche, Delbare, and Roels et al.^30^ also found the calibration of Airly LCSs using raw sensors’ output, and the reference-equivalent measurements will correct the sensor output with adequate compensation for potential temperature and RH effects.

**Figure 2:**
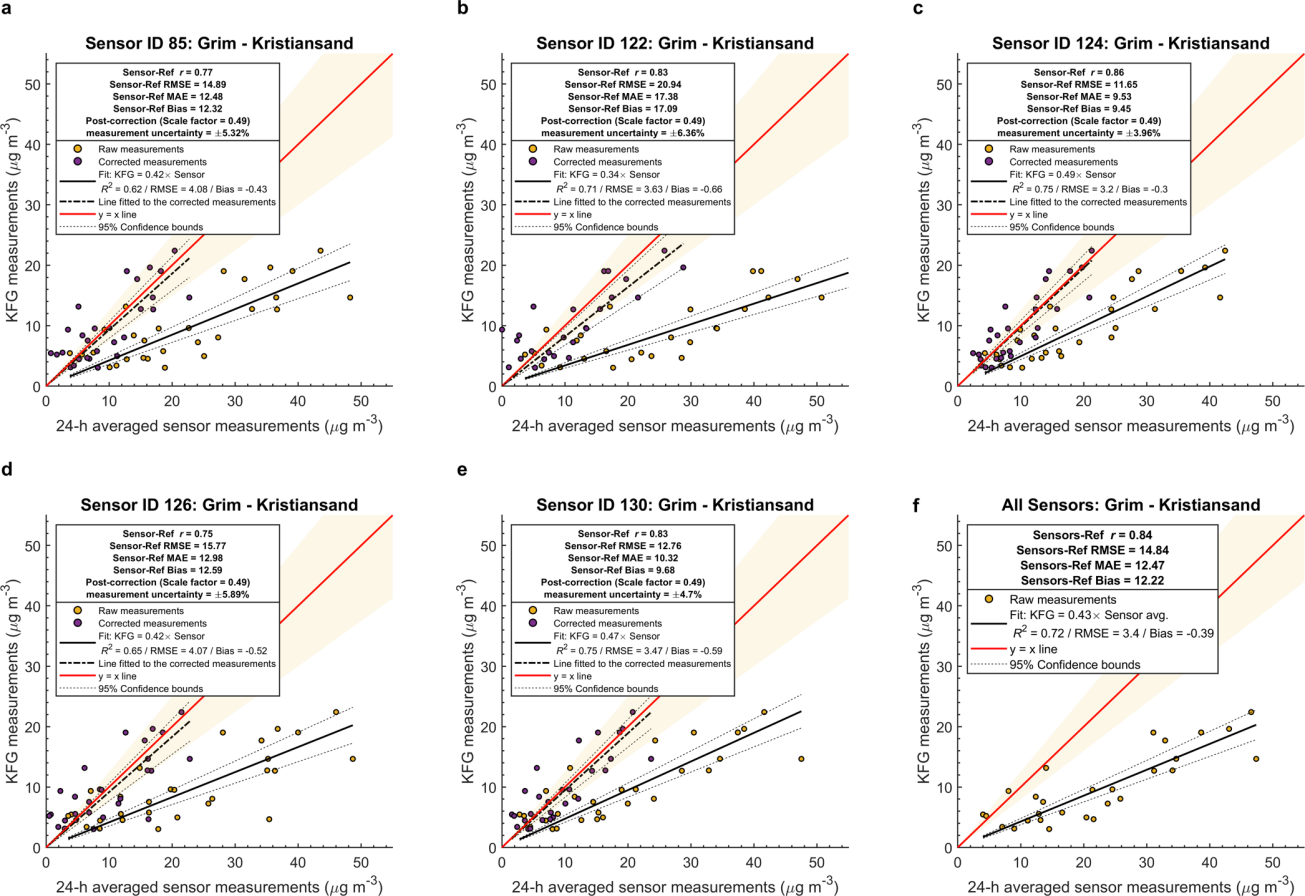
Comparison of 24 h averaged raw PM_2.5_ measurements of sensors with the corresponding gravimetric reference method (Kleinfiltergerät) measurements for two periods of 14 days—Jan 21, 2021 until Feb 03, 2021 and Feb 17, 2021 until March 02, 2021 (Kristiansand, Norway). (a–e) Individual sensors located in the Grim. Only sensor ID 124 was colocated in the garden of one of the households with KFG instrument; (f) mean of 24 h averaged measurements of all sensors located in the Grim neighborhood (sensors 85, 122, 124, 126, and 130). We have used iteratively reweighted least-squares fitting (robust linear regression) using the “bisquare” (“biweight”) weight function with the default tuning constant of 4.685 to reduce the outlier effect. For further details, the readers are referred to the MATLAB “fitlm” documentation (https://uk.mathworks.com/help/stats/fitlm.html, retrieved in Dec 2022). *r*: Pearson correlation coefficient. *R*^2^: coefficient of determination. RMSE: root-mean-square error. MAE: mean absolute error. Sensor-ref *r*, RMSE, and MAE values shown in the legends are calculated based on factory-calibrated sensor outputs. The uncertainty of the measurements corrected by the correction approach is represented in the legend title for each panel. Measurement uncertainties are calculated following the CEN report “Air Quality—Approach to Uncertainty Estimation for Ambient Air Reference Measurement Methods” (CR 14377:2002E; https://standards.iteh.ai/catalog/standards/cen/f4b43420-4322-4bfa-abe5-642b7d47ff8a/cr-14377-2002). For each sensor, a normal probability distribution is fitted to the deviation of the corrected sensor measurements from the KFG device measurements. The lower and upper boundaries of the 95% confidence interval for the mean of the distribution were calculated. Finally, the percentages of measurement uncertainties were calculated by dividing the estimated confidence intervals by the “limit value” throughout the analysis. The European air quality directive 2008/50/EC (https://eur-lex.europa.eu/legal-content/EN/TXT/PDF/?uri=CELEX:32008L0050&from=en; see ANNEX II upper and lower assessment thresholds) suggests that a limit value of 24 μg m^–3^ for continuous measurements of PM_2.5_.

Figure 2c shows a scatter plot relating observations from the daily gravimetric measurements (KFG) against daily averages of the Airly LCS colocated in the same backyard. For PM_2.5_, the factory-calibrated output of colocated sensor ID 124 showed an MB of 9.45 μg m^–3^ and an RMSE of 11.65 μg m^–3^. The daily factory-calibrated PM_2.5_ measured by other sensors (Figure 2a,b,d,e) in the Grim area have an acceptable *r* with the reference data, with an *r* between 0.75 and 0.86. Note these results are relevant to winter time, and only sensor ID 124 was colocated with the KFG device; the maximum distance between the sensors and the KFG device was ≈487 m (sensor ID 85). For a summary of previous research on evaluating Airly PM LCS performance, please refer to Supporting Information SI.2.

Kang, Aye, Ngo, and Zhou^31^ reviewed 80 studies that evaluated the performance of PM_2.5_ LCSs in outdoor environmental settings and observed an  equal to 0.72 (Q1 = 0.53 and Q3 = 0.85) for PM_2.5_ ( = median *r*^2^, including *r* that were converted to *r*^2^). Among them,^26^ studies were focused on the evaluation of Plantower sensor kits; an  = 0.82 (Q1 = 0.65 and Q3 = 0.9) was observed against the reference-equivalent measurements. RMSE is less reported in the literature; however, Hong, Le, Tu, Wang, Chang, Yu, Lin, Aggarwal, and Tsai^32^ evaluated the hourly performance of 12 Plantower PMS5003 against BAM-1020 FEM (Beta Attenuation Monitor) reference-equivalent instrument over a 1–2 year period in Taiwan. They reported factory-calibrated RMSE values of 8.2, 15.53, 19.33, and 15.71 μg m^–3^ at different reference monitoring stations (RH varying between 70.5 and 100% and air temperature ranging between 22.8 and 36.5 °C). Correspondingly, Lee, Kang, Kim, Im, Yoo, and Lee^33^ colocated three Plantower PMS7003 at BAM PM711 High-End PM Monitoring Station in Seoul, Korea, from Jan 15, 2019 to Sept 4, 2019 and reported an average daily factory-calibrated RMSE of 17.3 μg m^–3^ (hourly RMSE = 22.05 μg m^–3^) for PM_2.5_ measurements. Overall, the error/MB and *r* values observed here align with the previous studies.

### 3.2 Comparison of Sensor Network and Reference Monitoring Stations

#### 3.2.1 Whole Period of Data Availability: Dec 2020–Aug 2022—Raw Data

The *r* between raw (before the 5-step data processing scheme) hourly PM_2.5_ measurements of each sensor and Bjørndalssletta and Stener Heyerdahl reference stations and the corresponding normalized-RMSEs (NRMSEs) during each month (Dec 2020–Aug 2022) are represented in Supporting Figures 8 and 9. RMSEs are normalized to the interquartile range of PM_2.5_ concentrations measured at the reference stations. Sensor data had to cover at least half of a month to be considered for calculating the *r* and NRMSE of that month.

Variations in microclimate across the city and hyperlocal sources of PM emissions can lead to substantial differences in measurements of optical PM sensors compared to reference stations.^34^ Based on the monthly *r*s between the raw output of sensors and the reference air quality stations, it is evident that the periods of April 2021 and April–May 2022 exhibit a significant disparity between the sensor readings and the official data. Spring cleaning activities increase variability across the city, causing increased differences between sensor measurements and reference stations.^35^ Kuula, Mäkelä, Aurela, Teinilä, Varjonen, González, and Timonen^36^ argue that the performance of PMS5003 in measuring PM_2.5_ depends on the stability of the ambient air size distribution; the rapid changes in ambient particle-size distribution and the proportions of mass in, for example, <0.8 and >0.8 μm fractions increase the PMS5003 inaccuracies. The increased disparity between the sensor’s output and the official measurements may also be attributed to the lower accuracy of the sensors, which could be influenced not only by high PM_2.5_ concentrations but also by the presence of high coarse PM.^37^

The data from two periods, Feb 2021 and March 2022, show the highest deviation of the sensors from the reference stations. According to the Copernicus Atmospheric Monitoring Service, Europe experienced PM pollution episodes during both periods. Between 19 and 27 of Feb 2021, a vast region of south and middle Europe and, to a lesser extent, north Europe were exposed to PM_10_ daily mean concentrations of 50–100 μg m^–3^ caused by an inflow of Saharan air along with significant dust.^38^ The Northern Europe PM levels were even stronger between 20 and 27 March 2022, driven by an extensive anticyclone associated with dry and stagnant conditions under a high-pressure system.^39^ According to the sensor reference manual (https://www.plantower.com/en/products_33/74.html, retrieved in Nov 2022; the error is ±10% at 100–500 μg m^–3^, while at 0–100 μg m^–3^, it is ±10 μg m^–3^) and previous studies, such as Kosmopoulos, Salamalikis, Pandis, Yannopoulos, Bloutsos, and Kazantzidis,^37^ Hong, Le, Tu, Wang, Chang, Yu, Lin, Aggarwal, and Tsai,^32^ and Kang, Aye, Ngo, and Zhou,^31^ uncertainties in PMS5003 measurements increase at higher concentrations of ambient PM_2.5_.

#### 3.2.2 During the Winters (Dec–Feb): 2020–2021 and 2021–2022—Corrected Data

The 5-step data processing scheme was applied to sensor network data during each winter. Our analysis mainly focused on the winter period. The measurement period includes the winters from 2021 (Dec 2020 to Feb 2021) and 2022 (Dec 2021 to Feb 2022). Only the sensors with at least 75% data coverage during either of these two winters or both were included in the analysis. Following the data correction/quality assurance, during the first winter, the average *r* between Stener Heyerdahl’s official measurements and the sensors’ PM_2.5_ measurements was 0.73. This quantity was 0.66 for the Bjørndalssletta reference station. The average RMSE values of sensors from the Stener Heyerdahl and Bjørndalssletta were 7.23 and 8.58 μg m^–3^, respectively. During the second winter (2021–2022), *r* values were 0.71 and 0.67, respectively, while RMSEs were 7.5 and 6.61 μg m^–3^ (Supporting Figures 10 and 11).

**Figure 3:**
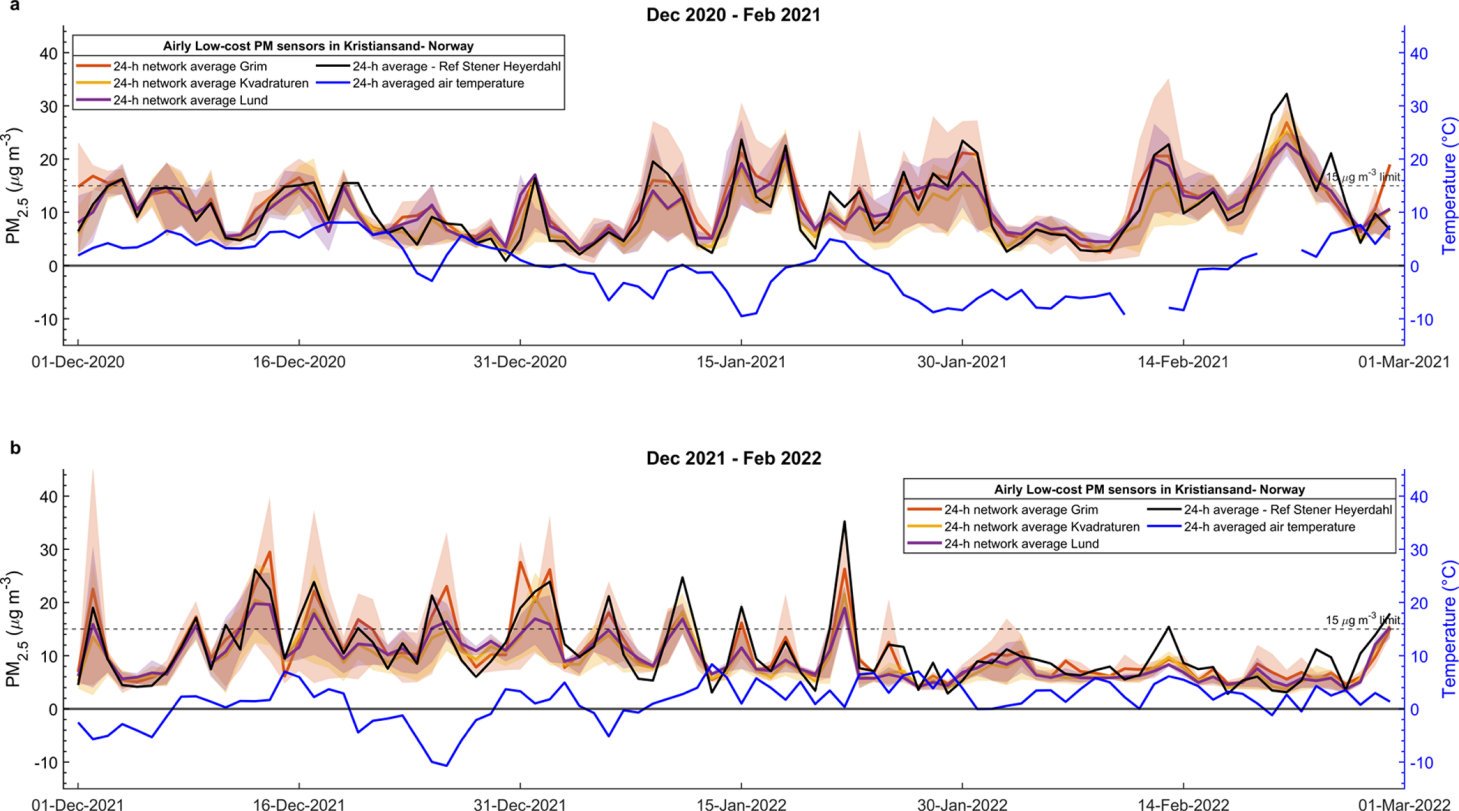
24 h average PM_2.5_ in different neighborhoods measured by a network of Airly low-cost particulate matter sensors during the winters of 2021 and 2022, Kristiansand, Norway. The filled areas represent the interquartile range of the daily averages recorded by individual sensors located within a neighborhood. The horizontal dotted line denotes the upper limit for the air quality threshold for health protection (24 h average PM_2.5_) (https://luftkvalitet.miljodirektoratet.no/artikkel/artikler/helserad_og_forurensningsklasser/, retrieved in Nov 2022; Norwegian Environment Agency). Supporting Figure 4 shows the maximum, minimum, and average daily temperatures for both winters obtained from the official meteorological station at the Kjevik airport, approximately 17 km northeast of Kristiansand. 2021 was a cold winter, with long periods of negative average daily temperatures and minimum temperatures reaching values below −10 °C. Kristiansand’s meteorological data during the analysis period were retrieved from the integrated surface data set (global) of the National Centers for Environmental Information (https://www.ncei.noaa.gov/access/search/data-search/global-hourly, accessed in Nov 2022) in the FM-15 surface meteorological airways format. The days with data coverage ≤ 75% were deleted from the analysis. With a mean daily average of 1.47 °C, winter 2022 was milder; however, there were also periods in December with negative daily average temperatures and minimum temperatures below −10 °C. The data after applying the five-step data processing scheme are used.

The time series of daily averaged PM_2.5_ within each neighborhood and whole city against the reference monitoring stations are represented in Figure 3 and Supporting Figure 12, respectively. During the first winter, Stener Heyerdahl and Bjørndalssletta reference monitoring stations measured PM_2.5_ levels of 10.9 and 12.43 μg m^–3^, respectively. These values were 10.92 and 10.23 μg m^–3^ during the second winter. Overall, the daily PM_2.5_ concentrations in ambient air are higher during the first winter; this can be attributed to the colder winter of 2020–2021 (especially January onward) and the resulting higher RWC. The sensors tended to underestimate the PM_2.5_ concentrations at low ambient PM concentrations and overestimate at high ambient PM concentrations (Supporting Figure 13).

Supporting Figure 14 shows the diurnal cycle calculated by using the data from the LCSs and the data from the two reference stations. The diurnal variation of the sensor data is calculated as the mean of all deployed sensors per hour of day per sampling site. According to the results, two peaks in PM_2.5_ concentrations (bimodal distribution), one between 8:00 and 12:00 and the other between 16:00 and 23:00, can be observed in all three neighborhoods. The peak in the morning might be attributed to a lower boundary layer, fumigation effect^40^—which breaks nighttime inversion due to strengthened thermals after the sunrise and causes mixing downward of aerosols stabilized in the nocturnal residual layer—during early morning rush-hour traffic,^41,42^ and RWC (due to the Covid-19 lockdown). At the same time, the PM_2.5_ concentration reduction in the afternoon (12:00–16:00) is mainly associated with the higher boundary layer height^43^ and less biomass burning. A similar bimodal distribution has been observed in previous studies, such as Ravindra, Singh, Mor, Singh, Mandal, Bhatti, Gahlawat, Dhankhar, Mor, and Beig,^44^ Singh, Singh, Biswal, Kesarkar, Mor, and Ravindra,^42^ and Yadav, Sahu, Beig, Tripathi, and Jaaffrey.^45^ The lowest concentrations of PM_2.5_ in all neighborhoods are observed during the early morning hours (4:00–8:00) in all regions.

The time series of the factory-calibrated output of the sensors during the two winters are represented in Supporting Figure 15, where the Valley Canyon effect^46^ on excessive PM_2.5_ concentrations in the Grim neighborhood is evident. The average PM_2.5_ concentrations during the first winter (2020–2021) in the three districts of Grim, Kvadraturen, and Lund were 10.86, 9.46, and 10.05 μg m^–3^, while during the second winter (2021–2022), the averages were 8.92, 8.36, and 8.65 μg m^–3^, respectively.

A comparison of the PM_2.5_ output of sensors against the traffic count data evidently (Supporting Figures 16 and 17) shows that the local traffic load is not associated with the measured PM_2.5_ levels, as reported in some studies, e.g., ref (47). The daily averaged PM_2.5_ sensor measurements are compared with the total vehicles per day counted by official traffic inductive loops across the city. Traffic data were retrieved from Statens Vegvesen—The Norwegian Public Roads Administration (https://www.vegvesen.no/trafikkdata retrieved in Dec 2022).

### 3.3 High-Resolution Air Quality Mapping Using Sensor Data Assimilation

**Figure 4:**
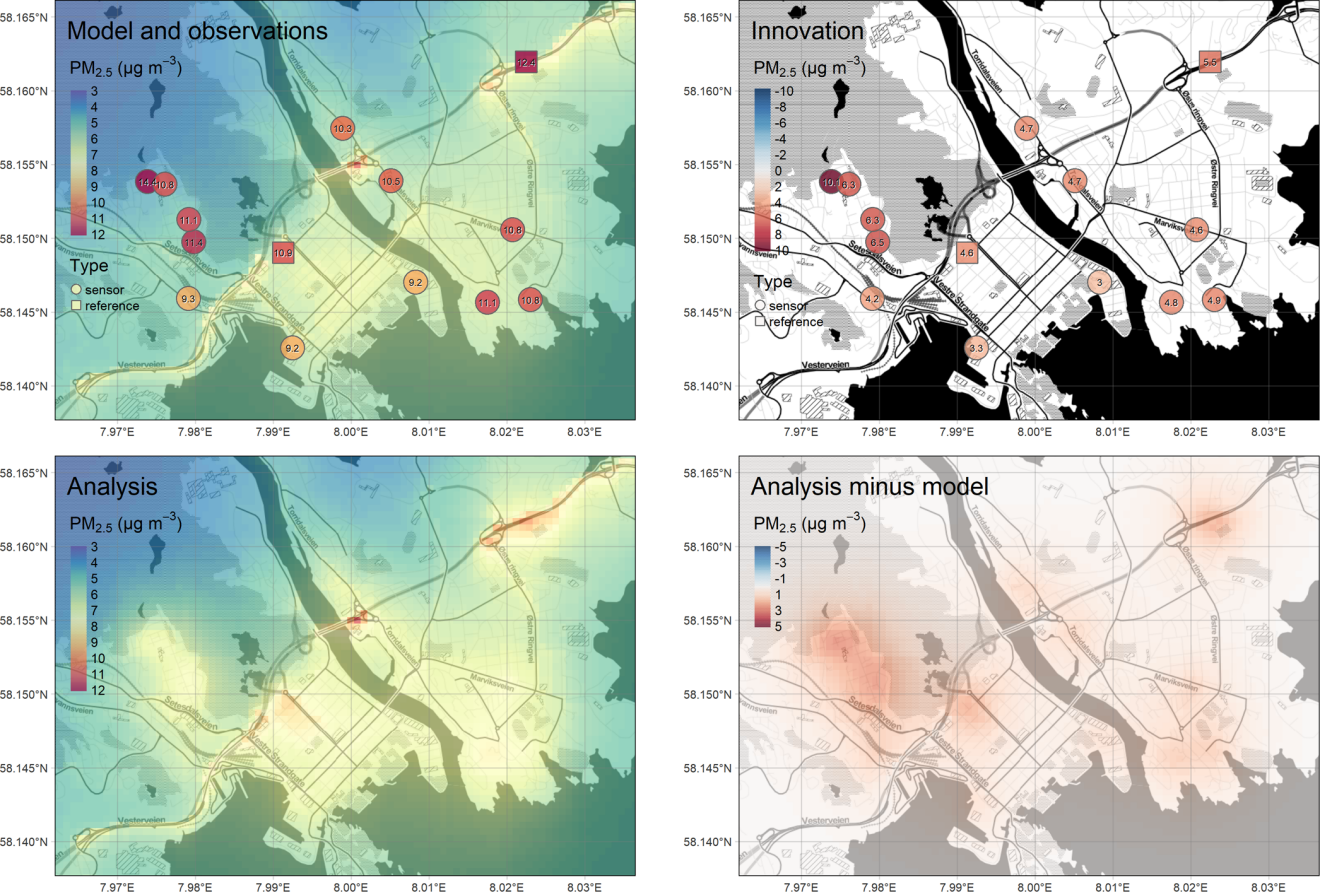
Combining observations of low-cost sensor systems with model information through data assimilation, here shown for PM_2.5_ for the period of 2020-12-01 through 2021-02-28. Top left panel: original uEMEP model, a priori data set (background), and sensor observations (symbols); top right panel: the innovation, i.e., the difference between model prediction and sensor observation, at the sensor deployment sites; bottom left panel: the concentration field resulting from the data assimilation (the “Analysis”) and the original sensor observations; bottom right panel: difference between analysis and uEMEP model, indicating the spatial patterns of the corrections that were carried out as part of the assimilation. Base map copyright OpenStreetMap contributors and map tiles by Stamen Design, under CC BY 3.0. The data after applying the five-step data processing scheme are used.

The maps in Supporting Figure 18 show the spatial distribution of averaged PM_2.5_ concentrations measured by the static LCSs during the two winters of 2021 and 2022 against the corresponding output of the uEMEP air quality model. The maps show that the model cannot capture the high PM_2.5_ concentrations stemming from the RWC in the Grim area. Additionally, the model output for winter 2022 approximates a higher PM_2.5_ concentration, while the output of LCSs shows higher PM_2.5_ levels during the first winter (2021). We applied a simple OI-based data assimilation approach to combine the Airly PM LCS network observations with model information at an average seasonal scale. Figure 4 and Supporting Figure 19 show how data assimilation can be used with LCS data to update a modeled concentration field for PM_2.5_ using the original input data sets (uEMEP).

The assimilation results for the first winter period (2020-12-01 through 2021-02-28) are shown in Figure 4. The top left panel shows that the average sensor observations are generally higher than the model-predicted values (top left panel), particularly over the Grim area, where the sensors’ observations are significantly higher than the model-predicted values. This results in relatively high positive innovation values (top right panel). After the assimilation is carried out, the analysis (bottom left panel) shows correspondingly a significant positive adjustment of the model values over the Grim area. Both reference stations measured slightly higher values than the model predicted, so the values in the northwestern Kvadraturen area and around the Bjørndalssletta station have been moderately increased. The sensor-model differences were minor in the rest of the domain, and as such, the changes due to assimilation are more subtle in those areas.

Supporting Figure 19 shows the assimilation results for the second winter period (2021-12-01–2022-02-28). Once again, the sensor system deployed in the Grim area shows consistently higher PM_2.5_ values than those predicted by the model, although the overall levels are lower than those in the 2020–2021 season. Both reference stations and the rest of the sensor systems in the Kvadraturen and Lund areas show PM_2.5_ values slightly higher than those of the model. Correspondingly, the analysis shows an increase in PM_2.5_ levels around the Grim area by about 4–5 μg m^–3^. Similarly, the model values are corrected in the Kvadraturen and Lund areas, albeit only slightly by ca. 1–2 μg m^–3^. The results qualitatively reveal that data assimilation using a network of PM LCSs can improve the quality of high-resolution spatial maps of urban air quality.

We performed a leave-one-out cross-validation (LOOCV) scheme to assess the benefits of data assimilation. We ran the assimilation *N* times, with *N* representing the number of valid observations. In each run, one of the observations was excluded, and we compared the value of the assimilated map at the excluded site with that observed at the excluded site. This was repeated for all sites, resulting in *N* pairs of values (original model and assimilated map) for which the three summary metrics MB, RMSE, and MAE were computed across all *N* sites. This allowed us to evaluate the change in accuracy provided by the assimilation compared to that of the standard model run. We first report the LOOCV results for air quality monitoring stations equipped with reference instrumentation.

For the 2020–2021 season, the data assimilation results in a slight increase in the predicted PM_2.5_ of 6.93–6.98 μg m^–3^ at the Bjørndalssletta station and from 6.26 to 7.09 μg m^–3^ at the Stener Heyerdahl station. In both cases, the assimilation nudged the concentration field closer to the observed PM_2.5_ values of 12.4 and 10.9 μg m^–3^, respectively. For the 2021–2022 season, the values at the Bjørndalssletta station increased from 6.69 to 6.77 μg m^–3^ (observed value 10.2 μg m^–3^) and for the Stener Heyerdahl station from 6.14 to 7.91 μg m^–3^ (observed value 10.9 μg m^–3^). In all cases, these changes are in the right direction (toward the observed values) but relatively minor due to the stronger weight given in the assimilation to the highly accurate reference measurements.

However, the LOOCV results for the entire network show a much more significant impact of the assimilation: the results calculated over all observation sites (air quality monitoring stations and sensors) demonstrate that by assimilating the data from the sensor network and the two reference stations, we reduced the MB in the winter season 2020–2021 from 5.2 μg m^–3^ in the regular model run to 2.9 μg m^–3^ in the assimilation. The RMSE decreased from 5.5 to 3.4 μg m^–3^, and the MAE decreased from 5.2 to 3.1 μg m^–3^. This equates to relative accuracy improvements of approximately 44, 38, and 41% for MB, RMSE, and MAE, respectively. For the winter season 2021–2022, the MB reduced from 4.0 to 1.7 μg m^–3^ (−56%), the RMSE from 4.25 to 2.2 μg m^–3^ (−50.48%), and the MAE from 4.0 to 1.9 μg m^–3^ (−52%).

### 3.4 Limitations and Suggestions for Future Studies

- Facing a rapid increase in cases of the Covid-19 virus, the Norway government introduced a country-wide lockdown on March 12th, 2020, which lasted principally until May 7th, 2021. Similarly, during the second winter, a partial lockdown was imposed in Dec 2021 to respond to the Omicron variant of the virus. Thus, the results here are affected by measures imposed during the lockdown periods.
- The application of correction factors obtained from colocating one sensor with others may introduce uncertainties due to variations in sensor performance. Individual calibration of all sensors is the recommended quantitative solution to address this. However, in certain LCS studies with multiple sensors, calibrating each sensor individually may not be feasible due to time, funding, logistics, and human resource limitations.
- Only one sensor (ID 124) was colocated with the KFG device, while the remaining sensors were placed several hundred meters away. The spatial variability within the sensors (Grim area) could affect the sensor performance evaluation, even though they were deployed relatively close.
- The third step of the five-step data processing may lead to the exclusion of accurate sensor measurements. Quality control of crowdsourced data is challenging, but it is advisable to eliminate any erroneous values through basic checks.^48^ However, distinguishing between incorrect and correct data is not always definitive,^49^ which can limit the filtering approach as it may discard valuable data.
- The *r* between sensor measurements and filter samplers may vary depending on the type of aerosol being measured.
- The influence of RH was not explicitly considered during the sensor data processing. More advanced calibration techniques like machine learning can potentially incorporate RH in the calibration process. During both winters, the daily average for the RH was, most of the time, above 70% (87.22%).
- The uEMEP model has undergone extensive validation, but there may be instances where it disagrees with sensor data. Improvements to the model could involve refining emission inventories, enhancing the accuracy of emission factors, improving meteorological inputs (e.g., wind speed and direction), and enhancing the representation of chemical reactions.
- In this study, the adopted data assimilation approach only improved the spatial representation of the uEMEP model. Future research could explore the feasibility and benefits of assimilating data at an hourly resolution to better capture and represent system dynamics.
- Sensitivity analysis and comparison with existing models or assimilation techniques can help identify uncertainties and biases in the assimilation results.
- Collecting measurements that are not employed in the assimilation and original air quality models is advisable. These measurements should be included in future studies to validate the assimilation results.
- A potential suggestion is to consider using the nearest neighbor approach instead of the entire network during the data screening step 3. However, questions may arise about what criteria determine the nearest neighbors, such as spatial distance, similarity in PM emission sources, similarity in environmental conditions, or land use.

In conclusion, the study demonstrates the calibration and performance evaluation of Airly PM LCSs compared with reference monitoring methods. Pollution levels from PM_2.5_ were especially high in one of the neighborhoods (Grim) located in a small valley in the northern part of Kristiansand. The results indicate that including RH and intercept in the calibration equations may not be necessary for specific sensors/applications. Data proposed 5-step data correction/processing technique improved the accuracy of the sensor measurements, reducing the MAE and RMSE. The results also show that citizen observations using LCS can complement official in situ reference stations and air quality models, offering real-time high-resolution health protection data and evidence-based decision-making. The findings contribute to our understanding of the capabilities and limitations of LCSs for monitoring RWC.

## Data Availability

Sensor data required to replicate the results provided in this paper are available at the NILU’s sensor data platform, accessible at https://nordicpathlive.nilu.no/. Bulk data download requires a data access token that can be requested from the first/corresponding authors. Reference monitoring station data are available at https://luftkvalitet.nilu.no/en/historical.

## Supporting Information Available

Further details on the uEMEP air quality model, Airly sensors, gravimetric Kleinfiltergerät, and optimal interpolation approach are provided in the Supporting Information. (PDF)
