# Supplementary material for: Low-Cost Particulate Matter Sensors for Monitoring Residential Wood Burning
Source: Environ Sci Technol. 2023 Sep 27;57(40):15162–72. doi: 10.1021/acs.est.3c03661 (PMC10569052; doi:10.1021/acs.est.3c03661)
Supplement: Supplementary file 1 — es3c03661_si_001.pdf [file es3c03661_si_001.pdf]

Supplementary Information

Low-Cost Particulate Matter Sensors for Monitoring Residential Wood Burning

Amirhossein Hassan, Philipp Schneider, Matthias Vogt, and Núria Castell**
The Climate and Environmental Research Institute NILU
P.O. Box 100, Kjeller 2027, Norway.
* Correspondence: ahas@nilu.no, ncb@nilu.no

32 Pages
20 Figures
2 Tables

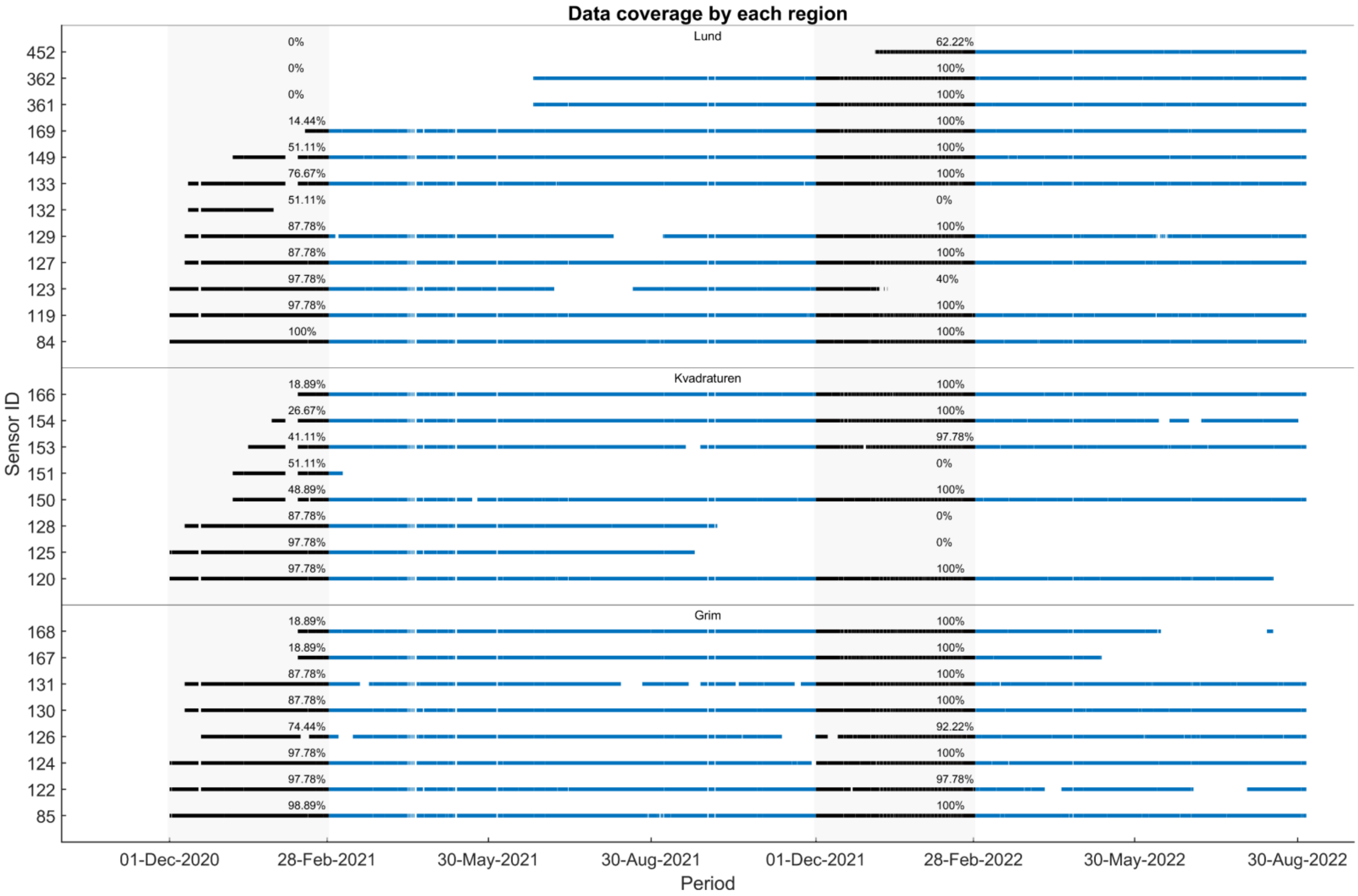

Supplementary Figure 1: Raw data coverage by individual Airly low-cost Particulate Matter sensors during the analysis period, Kristiansand, Norway. The grey zones represent the 2021 and 2022 winters. The percentage values shown for each sensor are the data coverage during that winter, calculated by dividing the number of days with full coverage by winter length (90 days). A day with at least 75% data coverage is assumed to be a full coverage day.

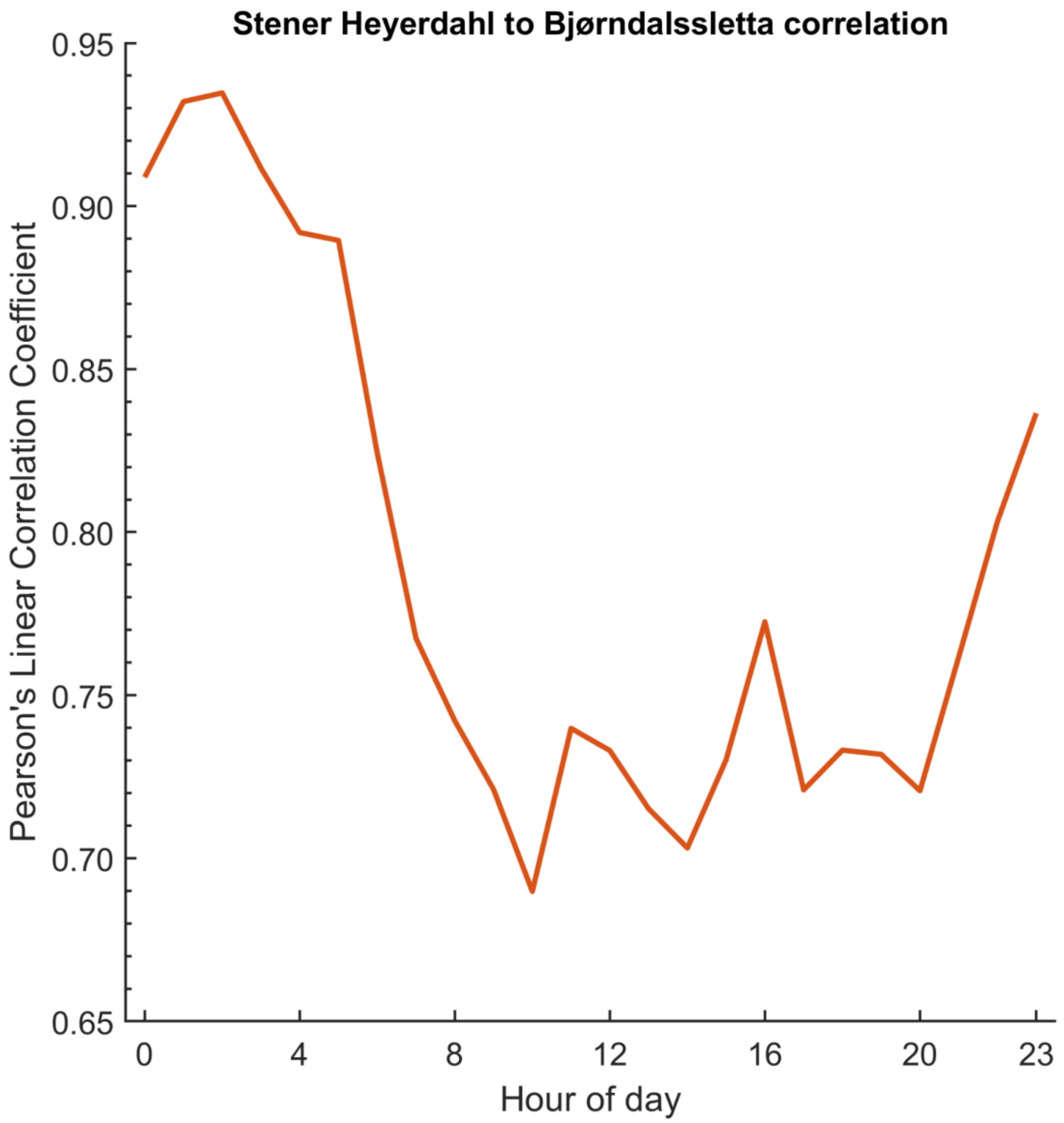

Supplementary Figure 2: The diurnal Pearson's Liner Correlation Coefficient between the reference air quality monitoring station $PM_{2.5}$ measurements, Kristiansand, Norway, 2020 - 2023 (winter local time).

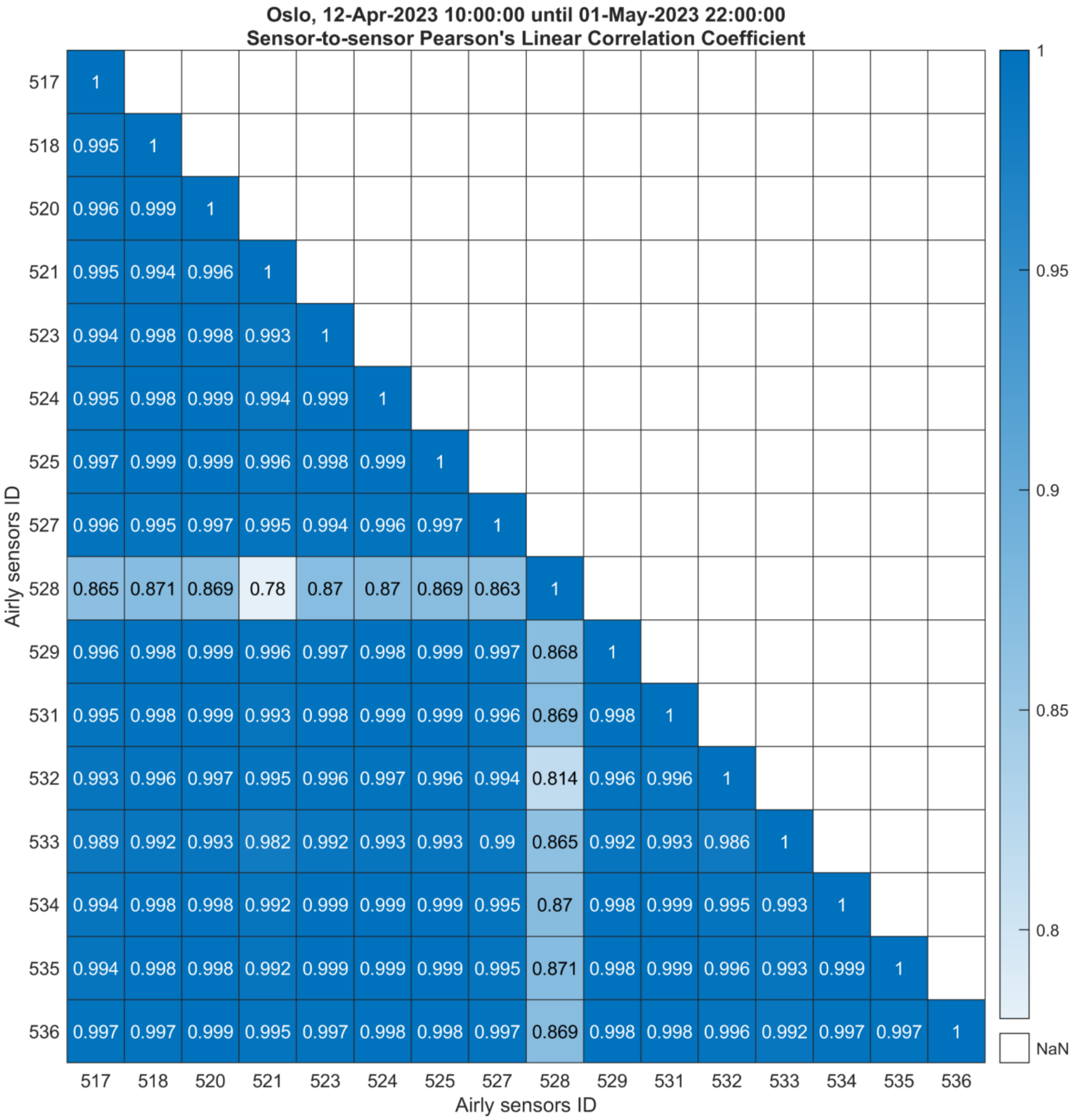

Supplementary Figure 3: Sensor-to-senor inter-comparison of 16 Airly PM LCSs, Oslo, Norway. The Values in each box represent the Pearson's Liner Correlation Coefficient between each pair of sensors' raw measurements (for 469 hours).

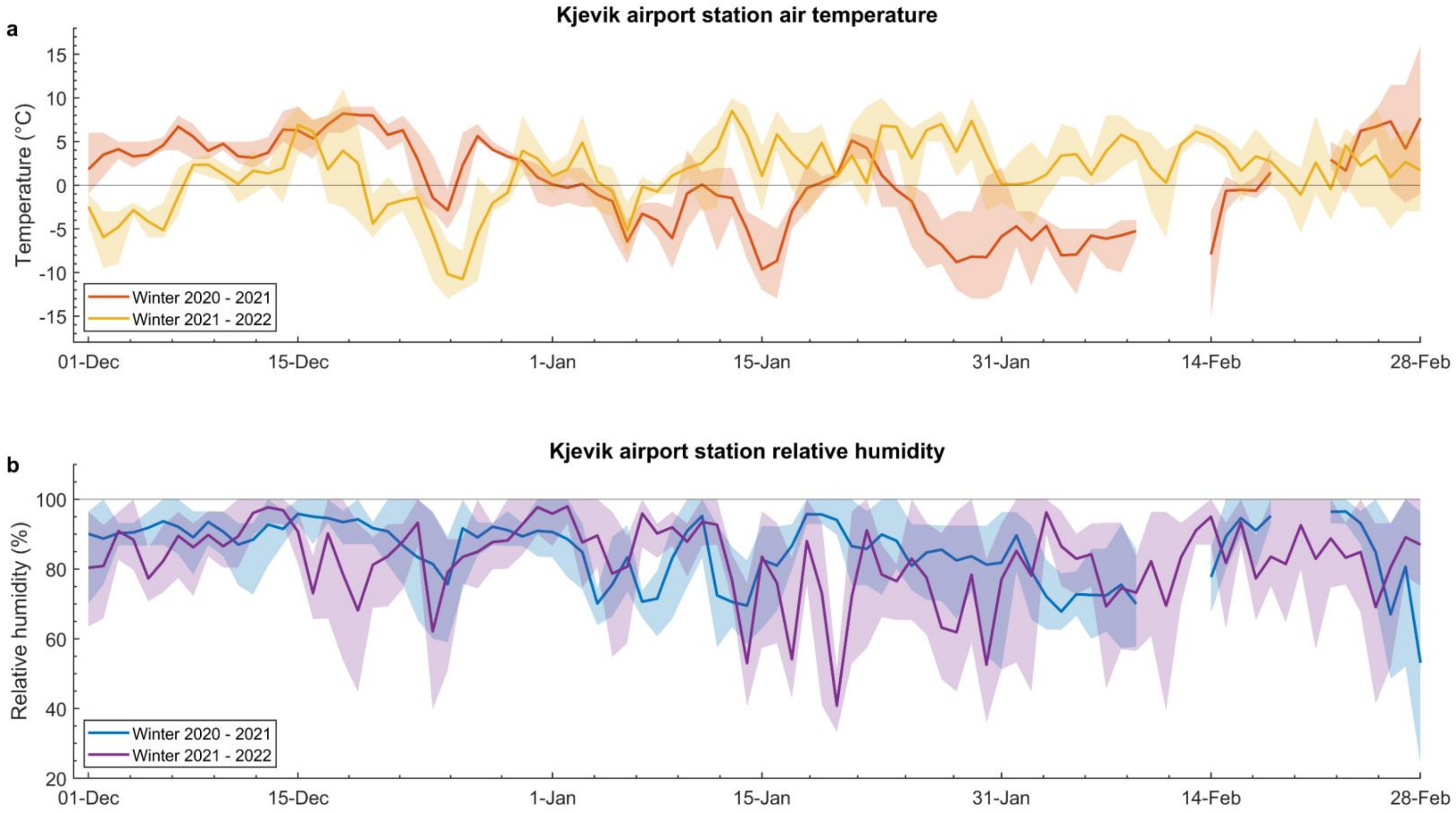

Supplementary Figure 4: Average daily air temperature and Relative Humidity measured at Kjevik airport station, Kristiansand, Norway, during the winters of 2021 and 2022.

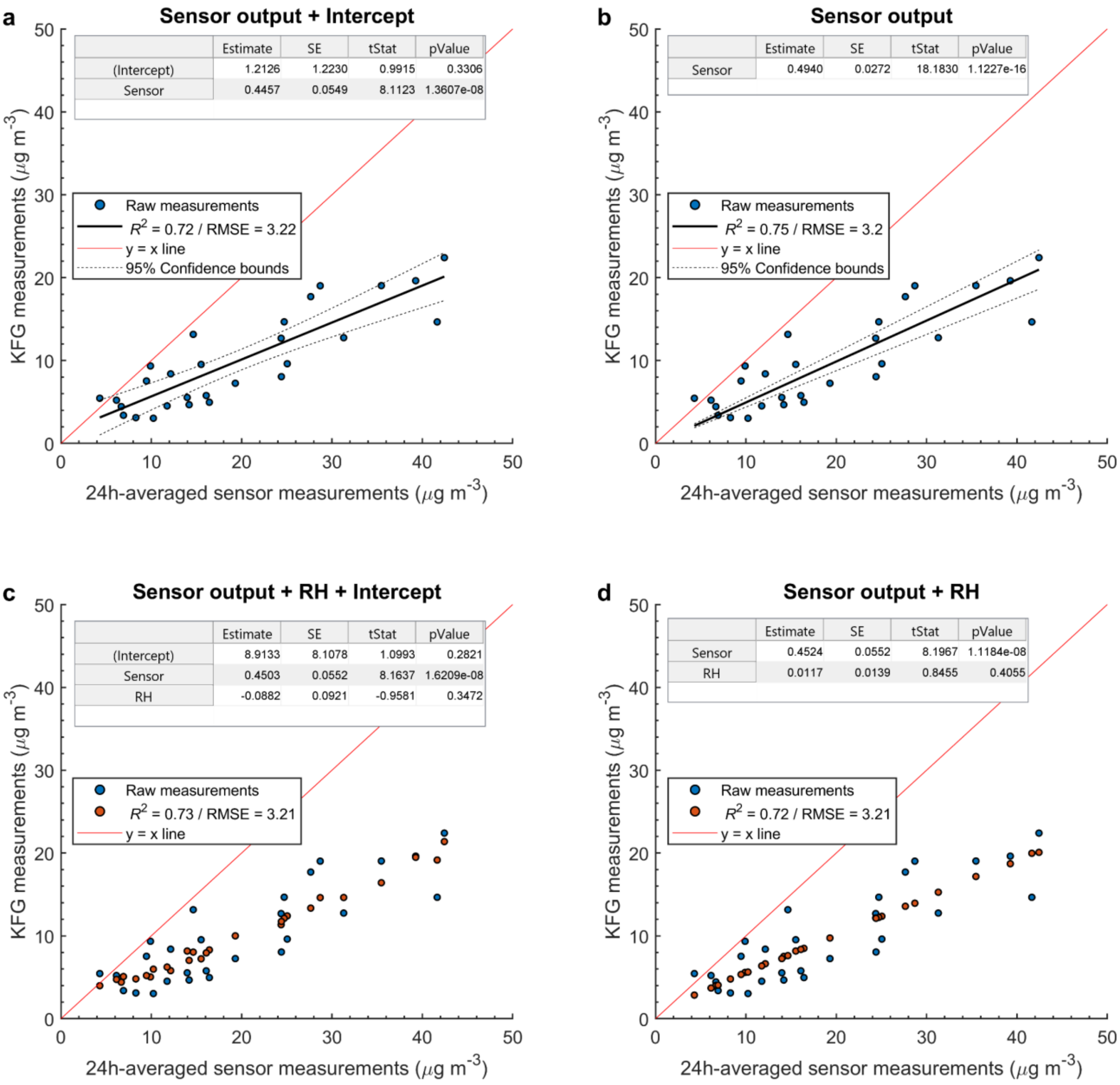

Supplementary Figure 5: Comparison of 24-h averaged raw $PM_{2.5}$ measurements of sensor ID 124 with corresponding gravimetric Small Filter Device (Kleinfiltergerät) measurements for two periods of 14 days — 21 January 2021 until 03 February 2021 and 17 February 2021 until 02 March 2021 (Kristiansand, Norway). The panel titles show the parameters used in the model for senor calibration. Estimate — Coefficient estimates for each related term in the model. SE — Standard error of the coefficients. t-statistic for each coefficient to test the null hypothesis that the corresponding coefficient is zero against the alternative that it is different from zero, given the other predictors in the model. Note that tStat = Estimate/SE. pValue — p-value for the t-statistic of the two-sided hypothesis test. For example, the p-value of the t-statistic for the intercept term in panel **a** is greater than 0.05, so this term is not significant at the 5% significance level given the other terms in the model.

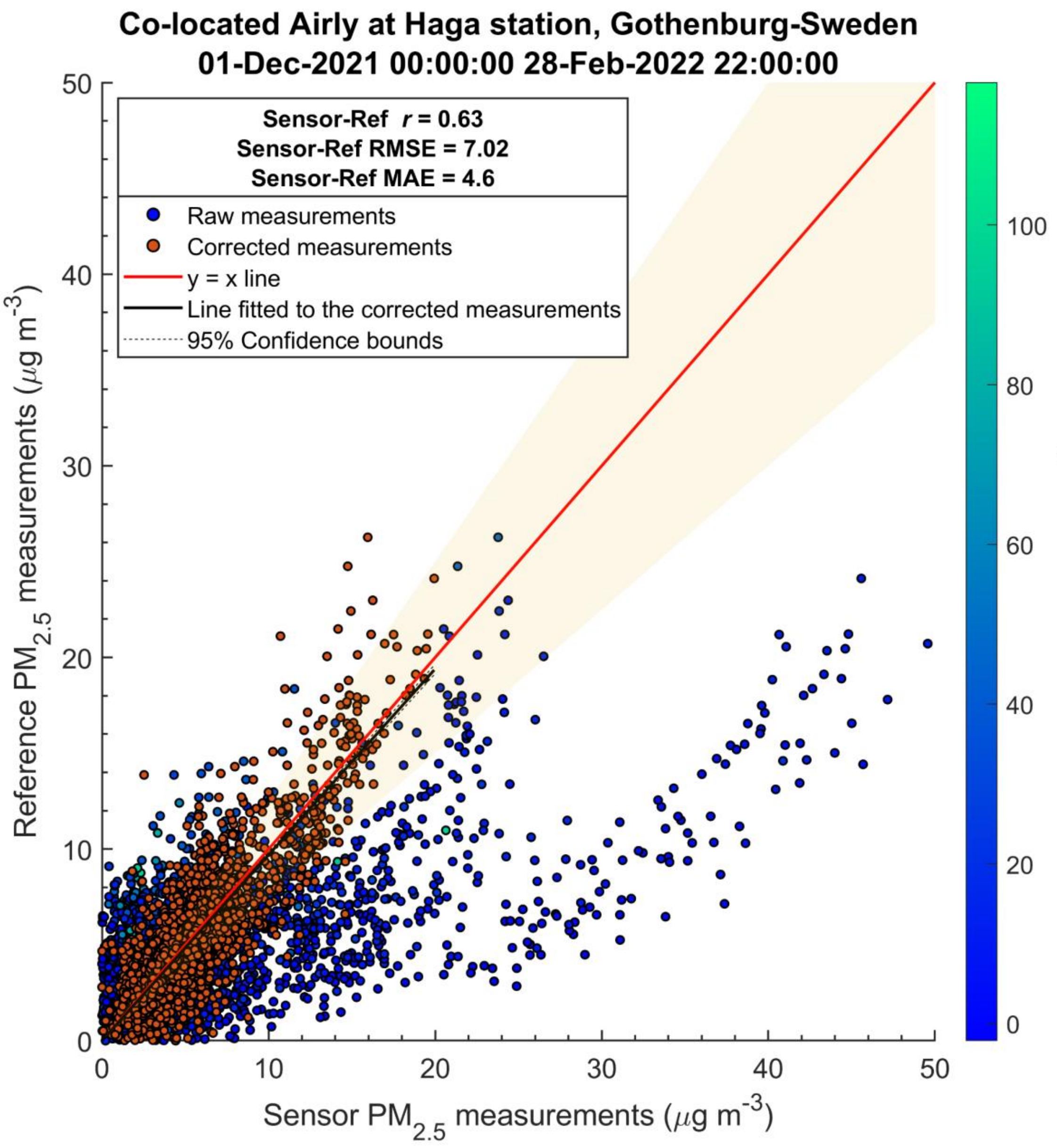

Supplementary Figure 6: Comparison of raw Airly sensor $PM_{2.5}$ measurements with the reference-grade optical instruments in Gothenburg. The filled yellow region highlights the areas between the y = 0.75x and y = 1.25x lines (25% uncertainty domain). The PM_{10} and $PM_{2.5}$ measurements used in the color bar for calculating coarse particle extent are obtained from the reference-grade instrument. The color scale in the figure represents the coarse particle fraction, with blue indicating lower coarse particle fractions and green showing higher fractions (PM_{10} minus $PM_{2.5}$). The unit used for the measurements is μg m^{-3}.

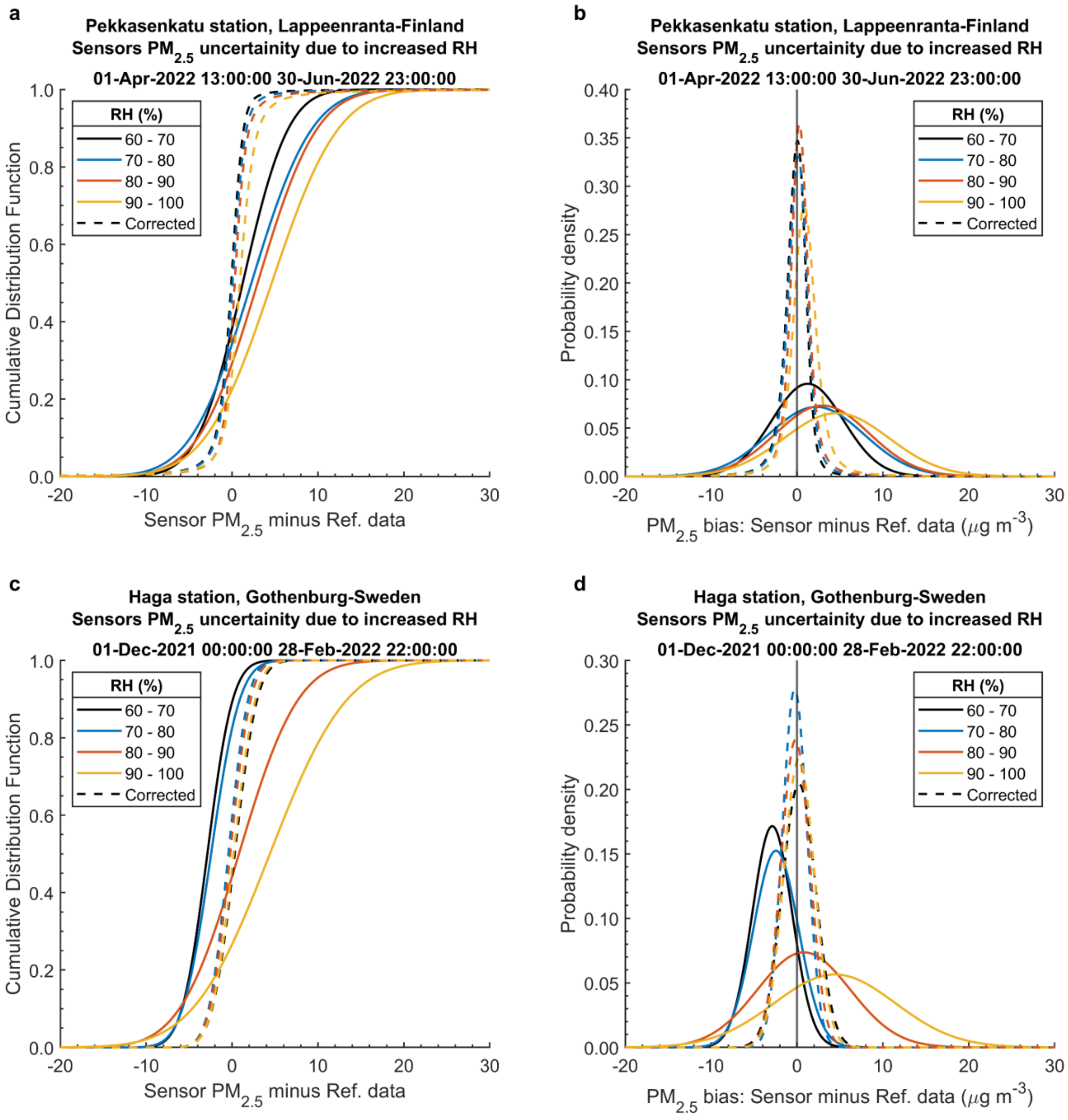

Supplementary Figure 7: An assessment of the Relative Humidity-related uncertainty of the Airly low-cost PM sensors. We fitted normal distributions to the sensor biases before and after correction at different RH bins (60:10:100%).

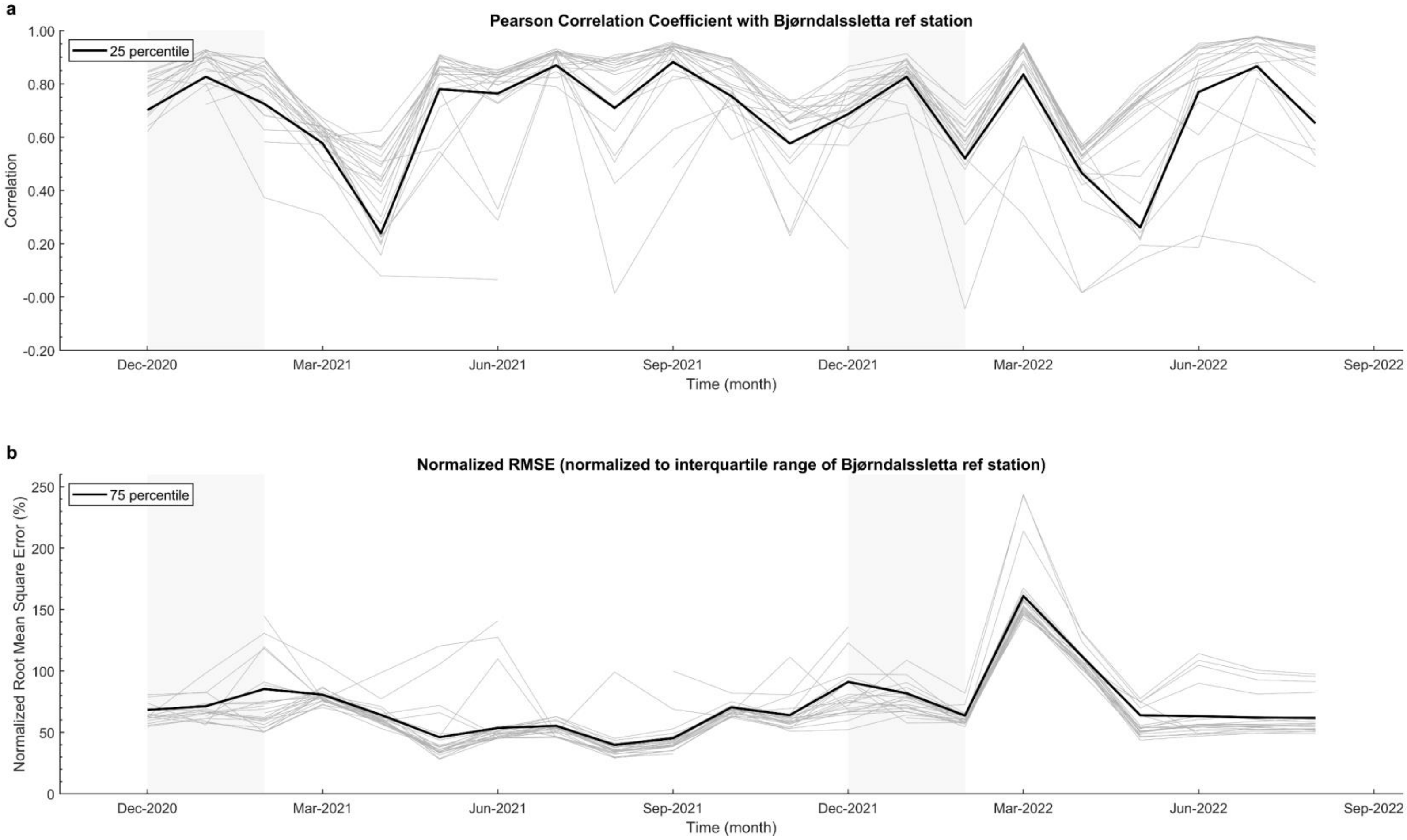

Supplementary Figure 8: Raw low-cost Airly sensor measurements of $PM_{2.5}$ against the *Bjørndalssletta* reference monitoring station in Kristiansand, Norway. a, Pearson Correlation Coefficient between sensors' measurements and station data. **b**, Normalized Root Mean Square Error of the sensors' measurements from station data. Values are normalized to the interquartile range of the official measured $PM_{2.5}$ during the data availability period (Dec 2020 – Aug 2022) in *Bjørndalssletta* station. Grey regions highlight the winter periods.

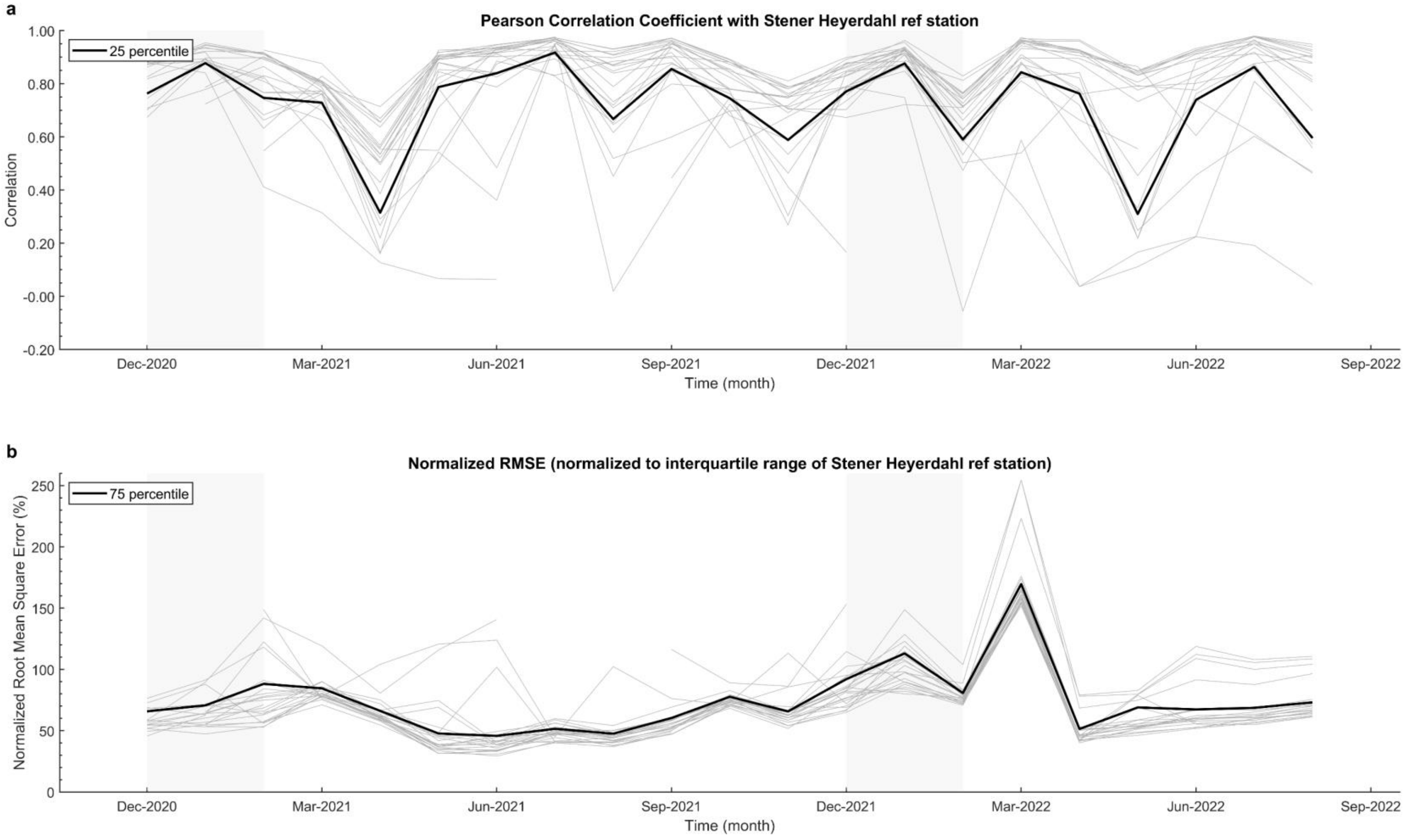

Supplementary Figure 9: Raw low-cost Airly sensor measurements of $PM_{2.5}$ against the *Stener Heyerdahl* reference monitoring station in Kristiansand, Norway. a, Pearson Correlation Coefficient between sensors' measurements and station data. **b**, Normalized Root Mean Square Error of the sensors' measurements from station data. Values are normalized to the interquartile range of the official measured $PM_{2.5}$ during the data availability period (Dec 2020 – Aug 2022) in *Stener Heyerdahl* station. Grey regions highlight the winter periods.

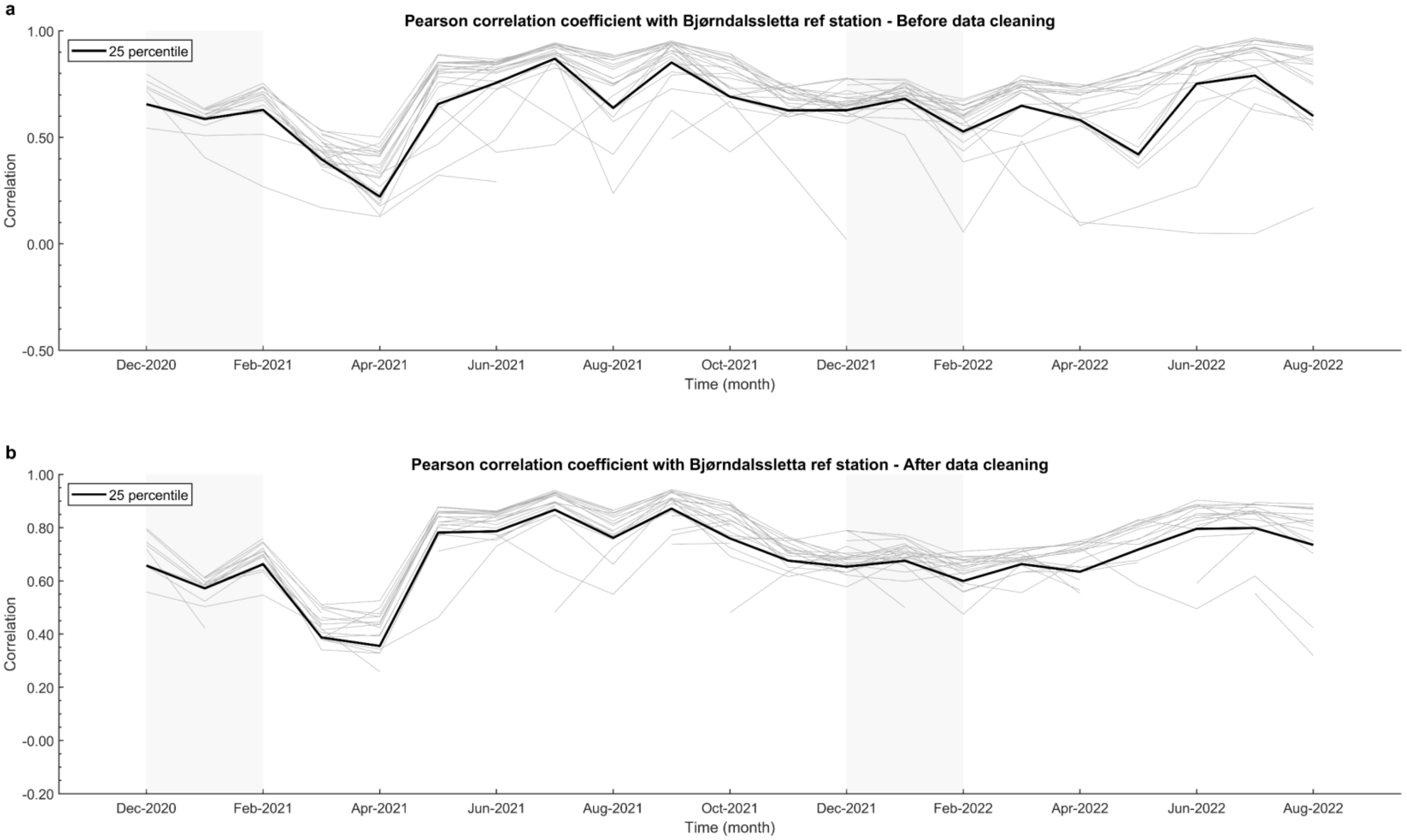

Supplementary Figure 10: Removal of out-of-range low-cost Airly sensors' measurements of $PM_{2.5}$, Kristiansand, Norway. a, Pearson Correlation Coefficient with *Bjørndalssletta* reference monitoring station before the third-step of the proposed five-step data processing. **b**, Pearson Correlation Coefficient with *Bjørndalssletta* reference monitoring station after applying the third-step of the proposed five-step data processing scheme. Grey regions highlight the winter periods.

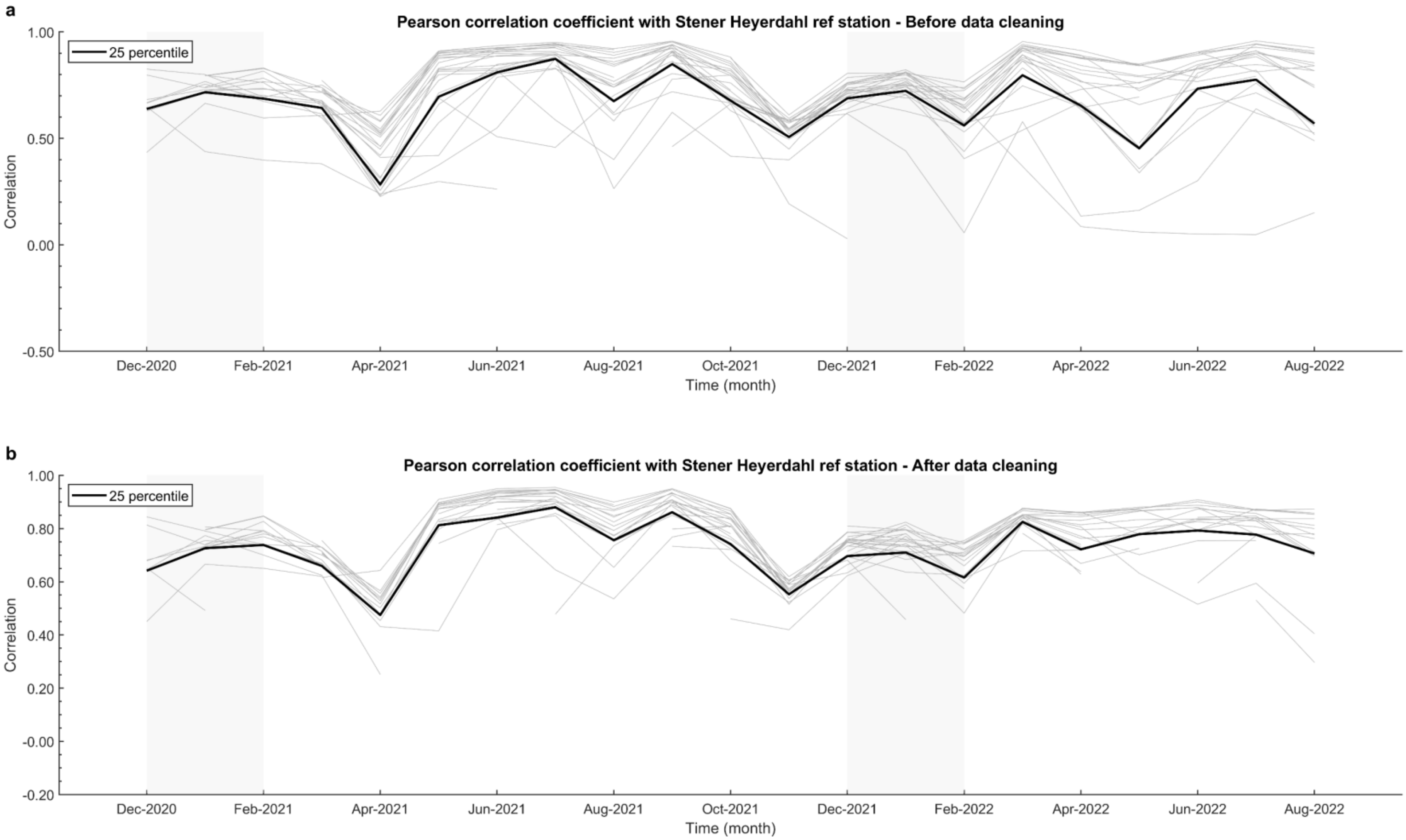

Supplementary Figure 11: Removal of out-of-range low-cost Airly sensors' measurements of $PM_{2.5}$, Kristiansand, Norway. a, Pearson Correlation Coefficient with *Stener Heyerdahl* reference monitoring station before the third-step of the proposed five-step data processing. **b**, Pearson Correlation Coefficient with *Stener Heyerdahl* reference monitoring station after applying the third-step of the proposed five-step data processing scheme. Grey regions highlight the winter periods.

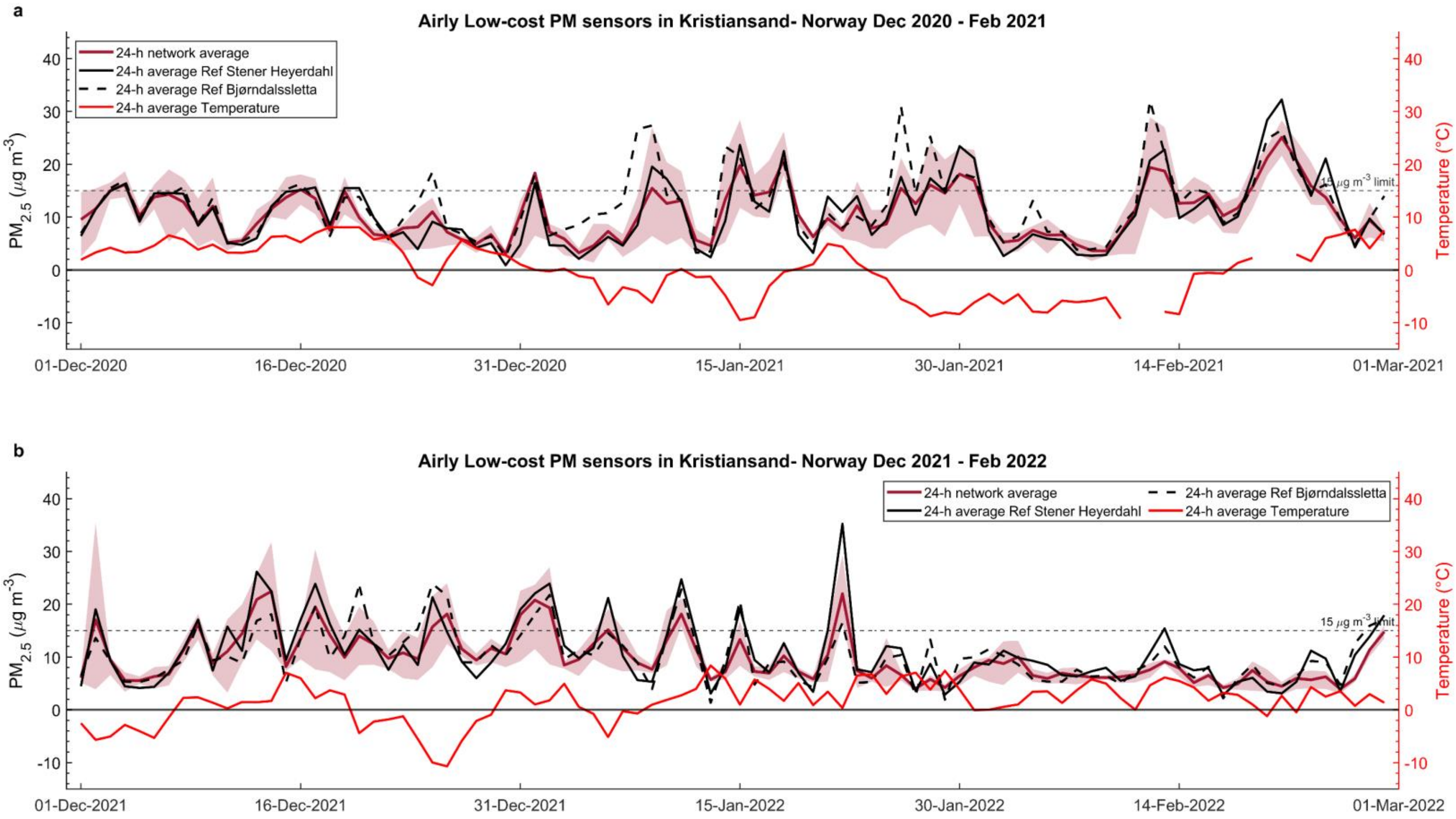

Supplementary Figure 12: 24-hour average $PM_{2.5}$ measured by a network of Airly low-cost Particulate Matter sensors during the winters of 2021 and 2022, Kristiansand, Norway. The filled areas represent the interquartile range of the daily averages recorded by individual sensors. The data after applying the five-step data processing scheme are used. The horizontal dotted line denotes the upper limit for the air quality threshold for health protection (24-hour average $PM_{2.5}$, https://luftkvalitet.miljodirektoratet.no/artikkel/artikler/helserad_og_forurensningsklasser/, retrieved in Nov 2022; Norwegian Environment Agency).

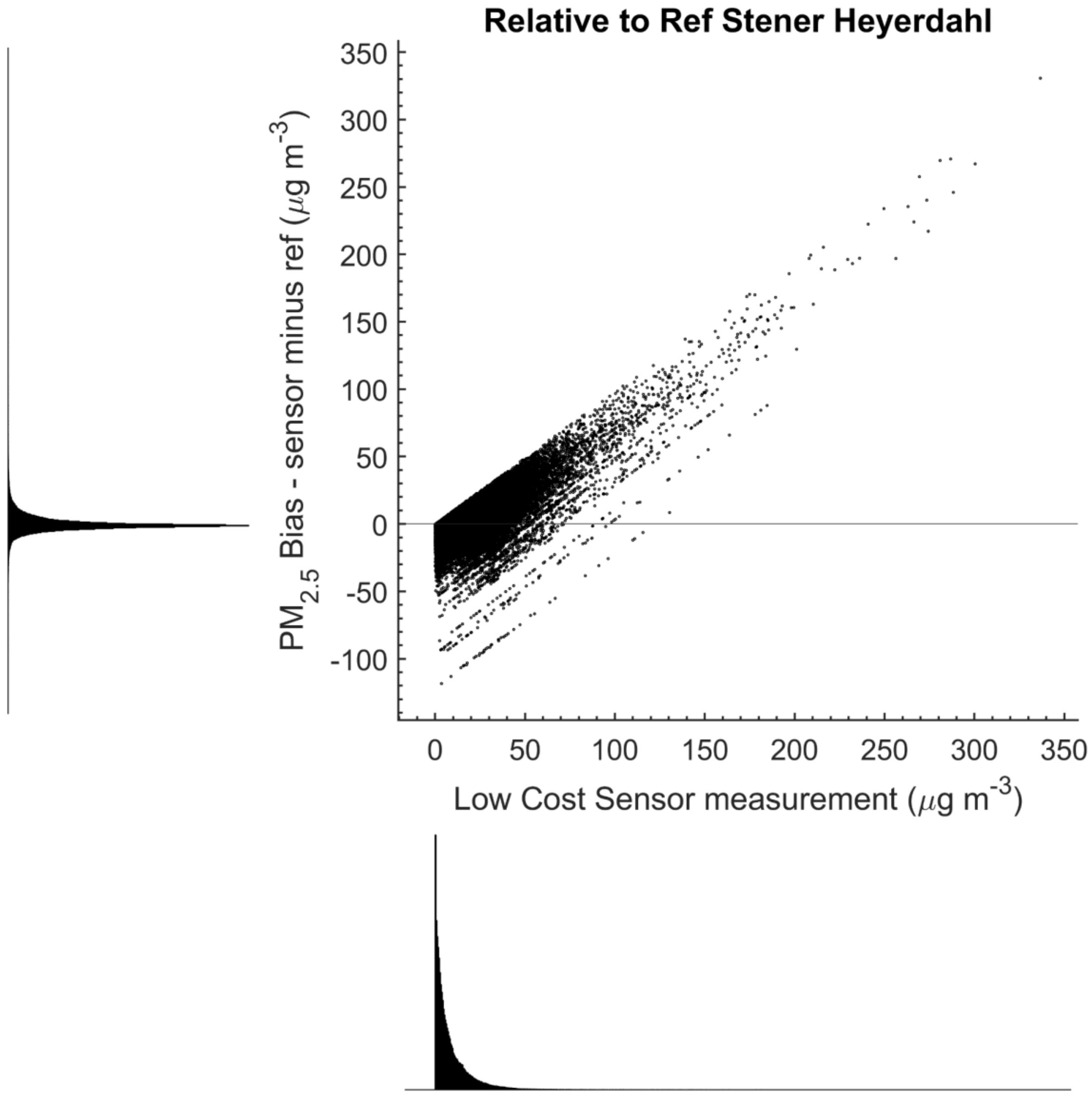

Supplementary Figure 13: Residual $PM_{2.5}$ (official *Stener Heyerdahl* station measurements – low-cost sensor measurements) against low-cost sensor measurements, Kristiansand, Norway. The whole data availability period is used. The raw data measured by sensors are used.

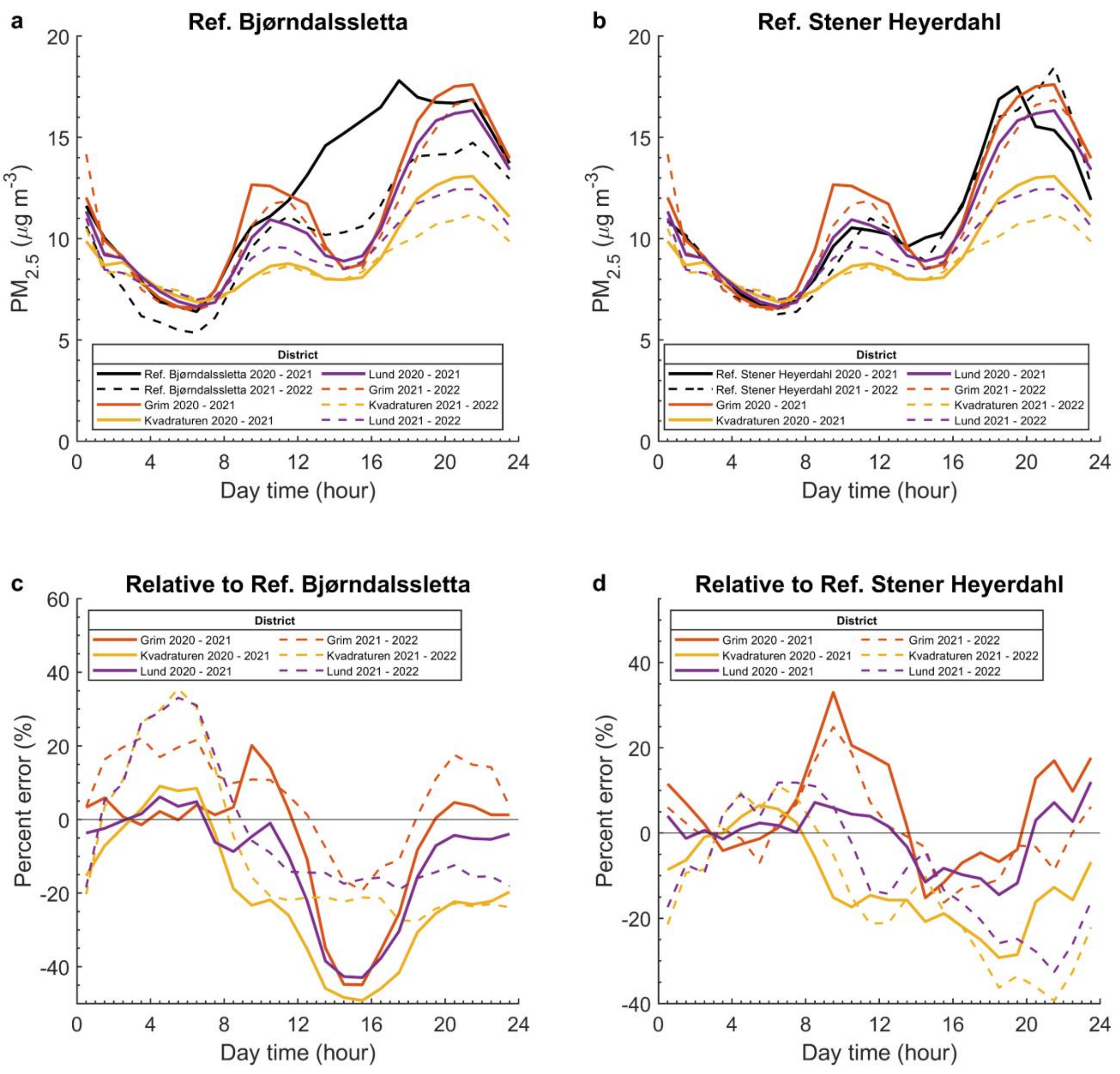

Supplementary Figure 14: Diurnal change in $PM_{2.5}$ in three districts (neighborhoods) of Kristiansand, Norway, measured by a network of Airly low-cost Particulate Matter sensors during the winters of 2021 and 2022. The panels also show the PM2.5 diurnal cycle for the two reference monitoring stations. **a** and **b**, absolute diurnal change compared to official measurements. **c** and **d**, relative diurnal changes calculated as *(Sensors Average – Ref Station)/ Ref Station × 100*. The data after applying the five-step data processing scheme are used.

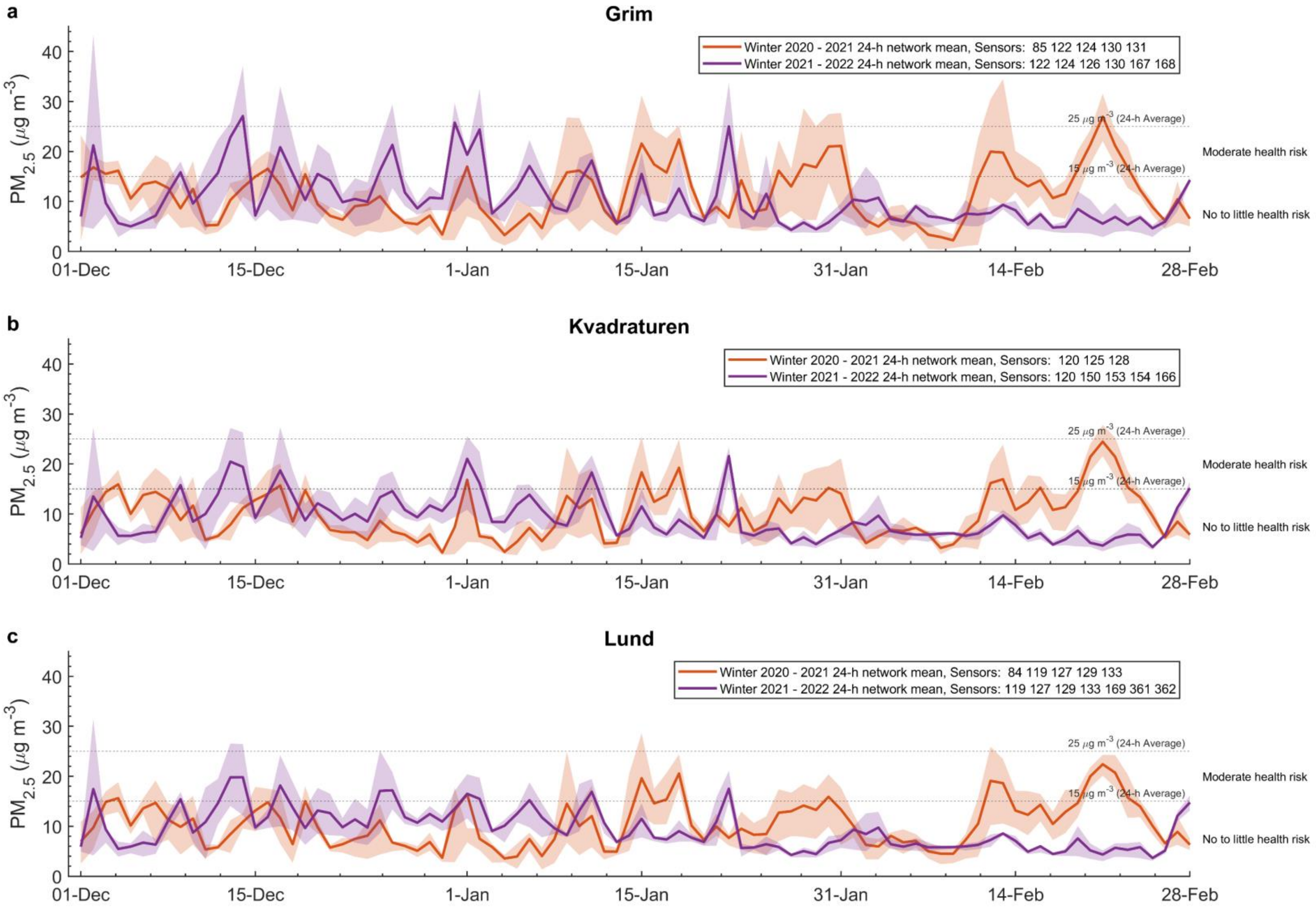

Supplementary Figure 15: 24-hour average $PM_{2.5}$ in different neighborhoods of Kristiansand, Norway, measured by a network of Airly low-cost Particulate Matter sensors during the winters of 2021 and 2022. The filled areas represent the difference between the minimum and maximum daily averages recorded by individual sensors located within a neighborhood. The data after applying the five-step data processing scheme are used. The horizontal dotted lines denote the upper limits for air quality thresholds for health protection (24-hour average $PM_{2.5}$, https://luftkvalitet.miljodirektoratet.no/artikkel/artikler/helserad_og_forurensningsklasser/, retrieved in Nov 2022; Norwegian Environment Agency).

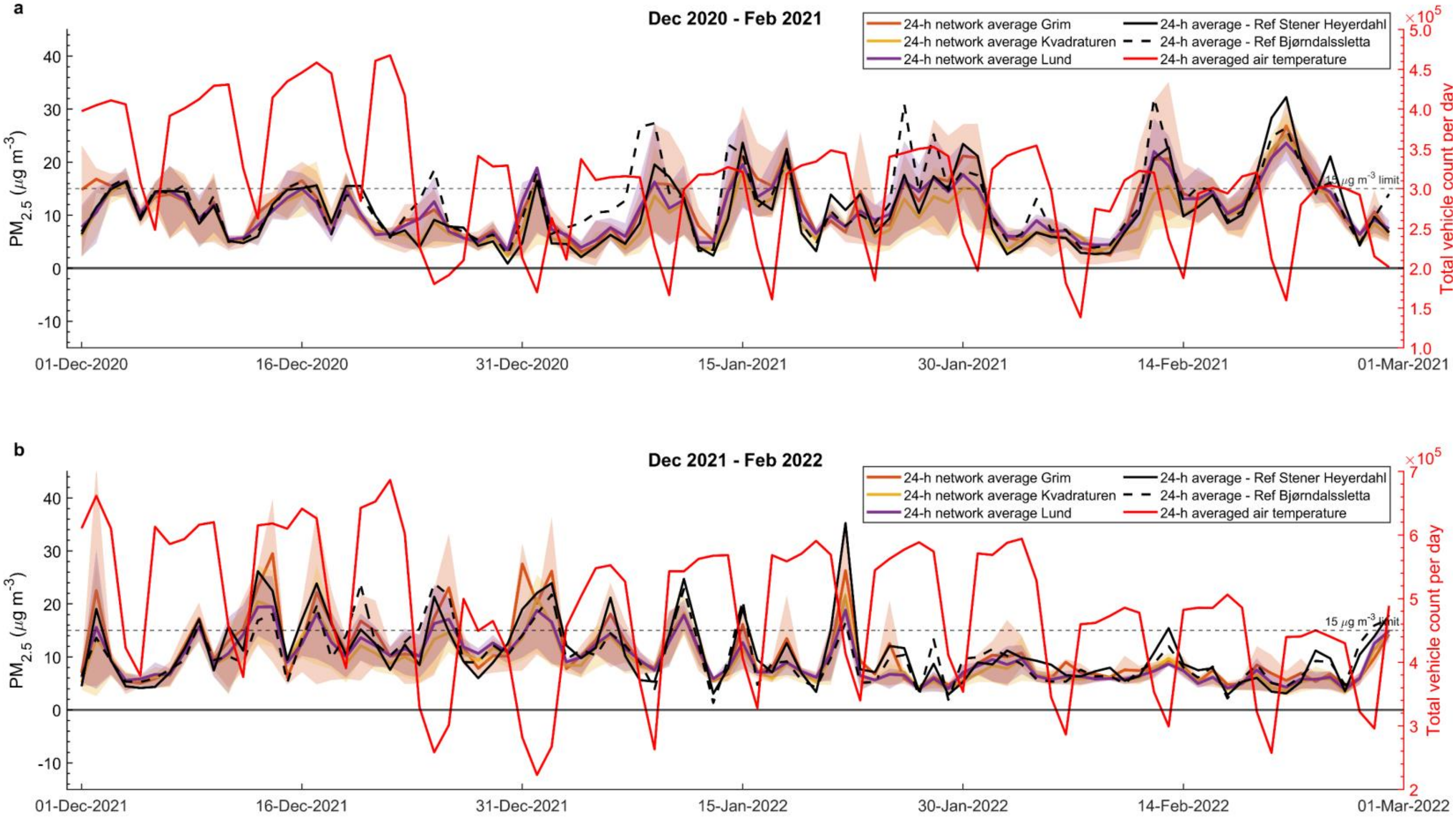

Supplementary Figure 16: 24-hour average $PM_{2.5}$ in different neighborhoods measured by a network of Airly low-cost Particulate Matter sensors during winters of 2021 and 2022, Kristiansand, Norway. The total vehicle count is the sum of counted vehicles measured at locations shown in Figure 1. The filled areas represent the difference between the minimum and maximum daily averages recorded by individual sensors located within a neighborhood. The horizontal dotted line denotes the upper limit for the air quality threshold for health protection (24-hour average $PM_{2.5}$, https://luftkvalitet.miljodirektoratet.no/artikkel/artikler/helserad_og_forurensningsklasser/, retrieved in Nov 2022; Norwegian Environment Agency). The data after applying the five-step data processing scheme are used.

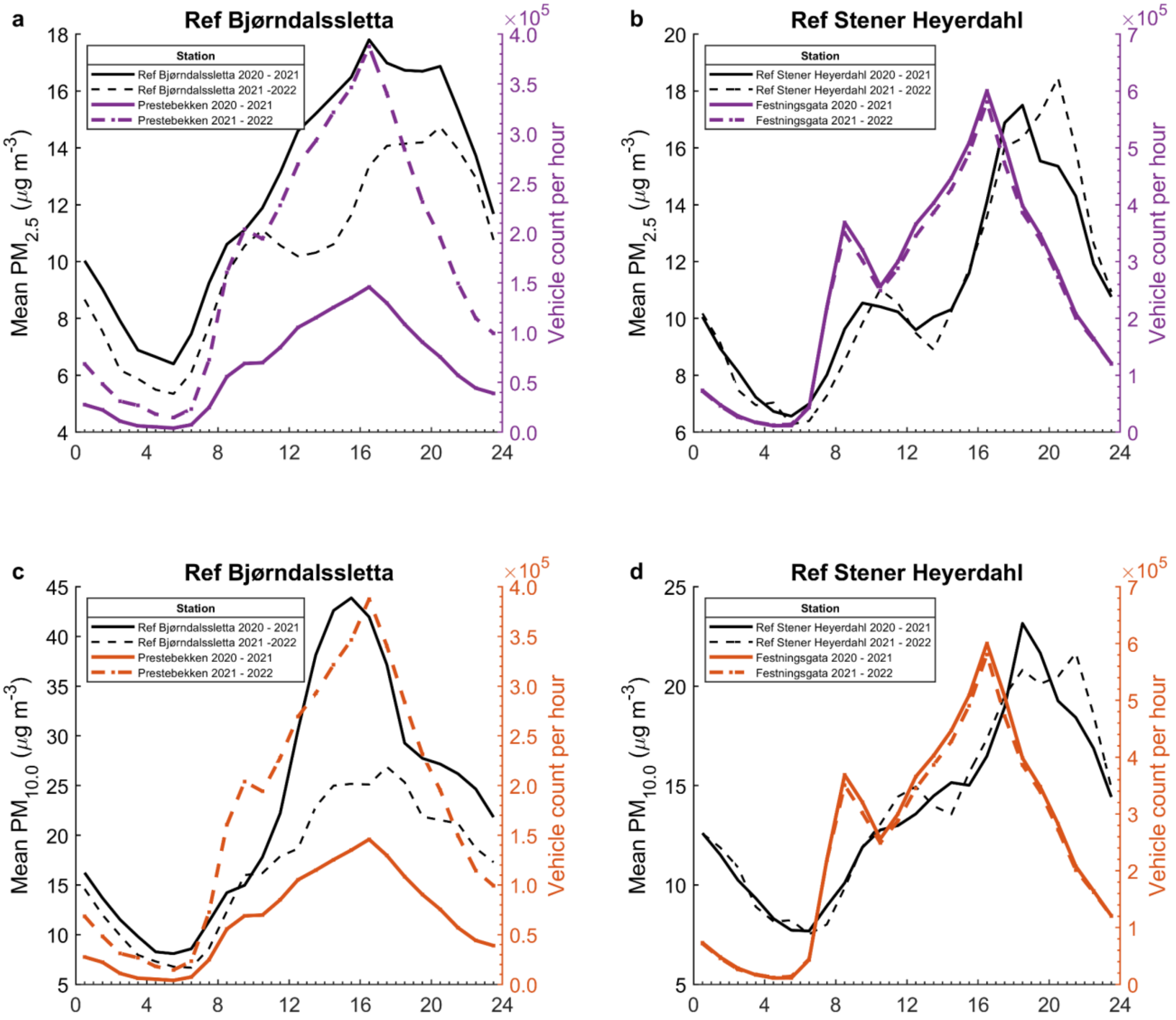

Supplementary Figure 17: Diurnal change in $PM_{2.5}$ in two reference monitoring stations and vehicle count at nearby Inductive Loops, Kristiansand, Norway. Data are relevant to the winters of 2021 and 2022. The locations of stations and Inductive Loops are represented in Figure 1. The data after applying the five-step data processing scheme are used.

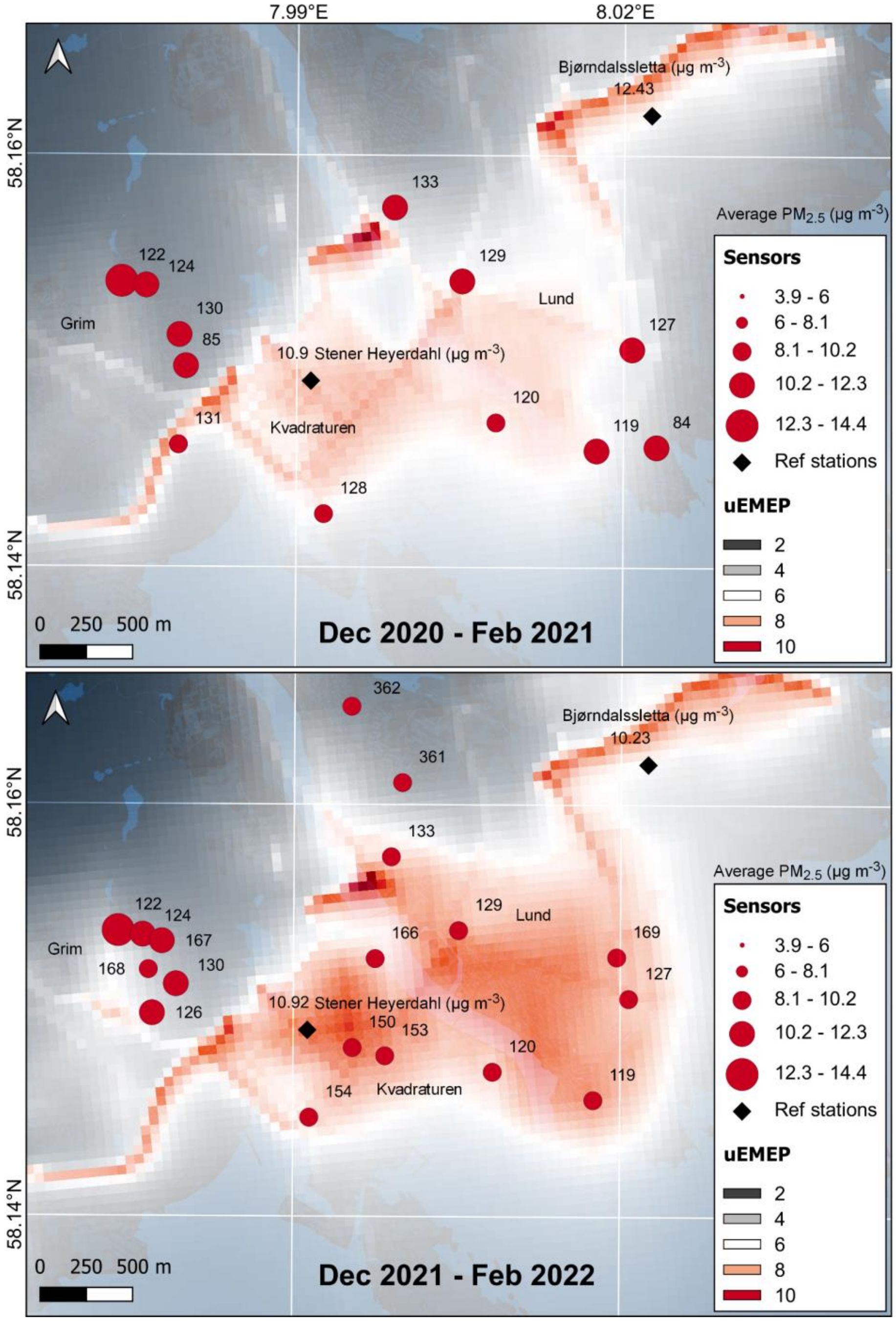

Supplementary Figure 18: Average $PM_{2.5}$ measurements of low-cost sensors during the two winters of 2021 and 2022 against uEMEP air quality model output in the background, Kristiansand, Norway. The data after applying the five-step data processing scheme are used.

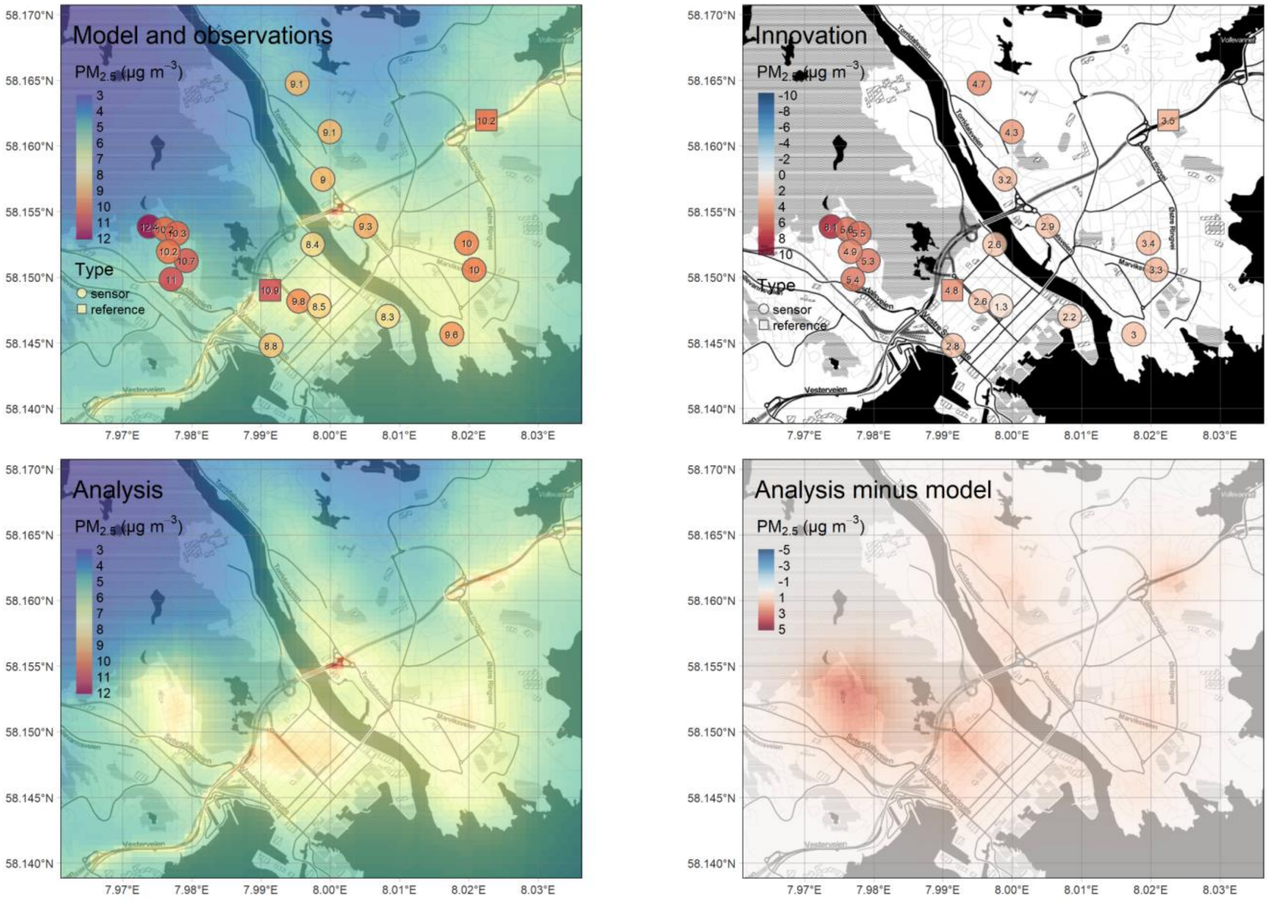

Supplementary Figure 19: Combining observations of low-cost sensor systems with model information through data assimilation, here shown for $PM_{2.5}$ for the period of 2021-12-01 through 2022-02-28. Top left panel: Original uEMEP model, a priori dataset (background), and sensor observations (symbols); Top right panel: The innovation, i.e., the difference between model prediction and sensor observation, at the sensor deployment sites; Bottom left panel: The concentration field resulting from the data assimilation (the "Analysis") and the original sensor observations; Bottom right panel: Difference between analysis and uEMEP model, indicating the spatial patterns of the corrections that were carried out as part of the assimilation. Base map copyright OpenStreetMap contributors and map tiles by Stamen Design, under CC BY 3.0. The data after applying the five-step data processing scheme are used.

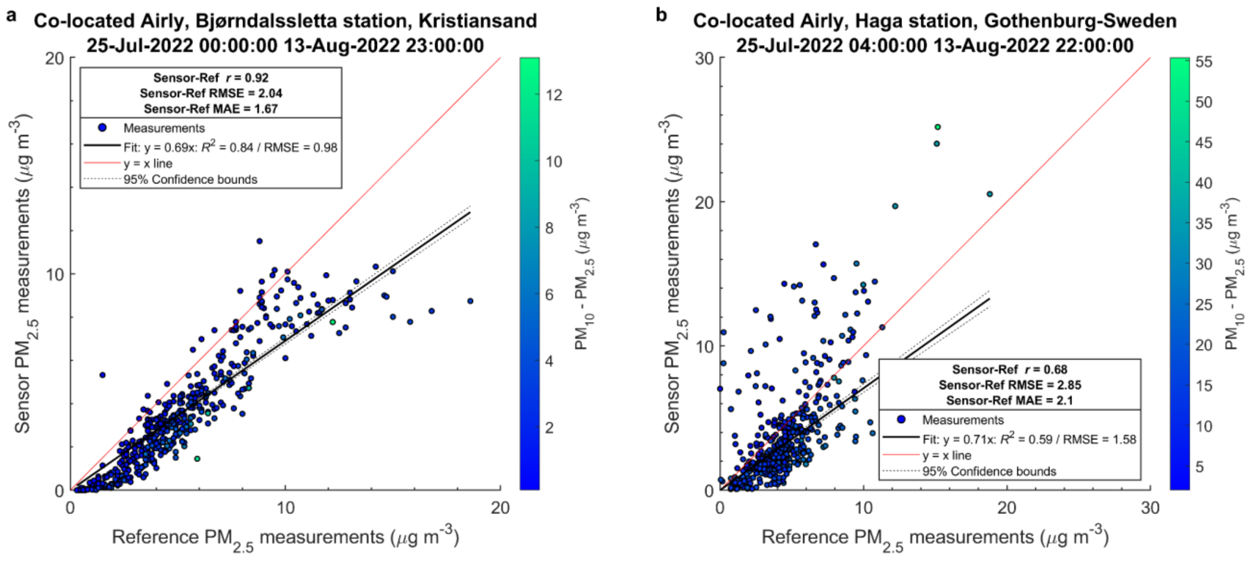

Supplementary Figure 20: Comparison of Airly sensor $PM_{2.5}$ raw measurements with the optical reference-grade optical instruments during summer. a, Kristiansand, **b**, Gothenburg.

Supplementary Table 1: Airly low-cost Particulate Matter sensors operated by citizens in Kristiansand, Norway. Sampling resolution: 3,600s. 28 of 30 sensors were analyzed in this study. Zone code 1 = Grim, Zone code 2 = Kvadraturen, and Zone code 3 = Lund.

id	Name	Altitude (m)	Created	Zone code
84	Airly_5548	8.5	2020-11-17T16:19:27.188694Z	3
85	Airly_5497	15.1	2020-11-17T16:21:00.941845Z	1
86	Airly_5761	15.3	2020-11-17T16:22:34.904432Z	5
119	Airly_6289	18.1	2020-11-30T14:37:26.613716Z	3
120	Airly_6211	1.9	2020-11-30T14:37:27.534309Z	2
122	Airly_6434	5.5	2020-11-30T14:37:29.515426Z	1
123	Airly_5863	27.4	2020-11-30T14:37:30.487448Z	3
124	Airly_6148	4.6	2020-11-30T14:37:31.460115Z	1
125	Airly_6471	7.7	2020-11-30T14:37:32.635938Z	2
126	Airly_6236	10.5	2020-11-30T14:37:33.591468Z	1
127	Airly_6205	7.2	2020-12-09T12:30:53.561377Z	3
128	Airly_6352	0.6	2020-12-09T12:30:54.683702Z	2
129	Airly_6220	4.1	2020-12-09T12:30:57.732633Z	3
130	Airly_6213	16.1	2020-12-09T12:30:58.764566Z	1
131	Airly_5490	15.2	2020-12-09T12:30:59.808829Z	1
132	Airly_5518	7.3	2020-12-11T09:38:24.128596Z	3
133	Airly_5515	7.4	2020-12-11T09:38:25.10758Z	3
149	Airly_5506	10.4	2021-01-05T14:15:10.166855Z	3
150	Airly_5558	7.0	2021-01-05T14:16:36.105813Z	2
151	Airly_5568	5.5	2021-01-05T14:16:59.254479Z	2
153	Airly_5503	6.4	2021-01-14T08:59:59.112319Z	2
154	Airly_6343	1.9	2021-01-27T16:04:49.417132Z	2
166	Airly_6216	3.3	2021-02-10T21:07:48.968974Z	2
167	Airly_6231	4.7	2021-02-10T21:08:08.73889Z	1
168	Airly_6279	4.7	2021-02-10T21:08:23.094037Z	1
169	Airly_6208	7.7	2021-02-15T10:07:44.135922Z	3
360	Airly_6159	55.6	2021-06-24T07:53:02.562762Z	5
361	Airly_6155	21.6	2021-06-24T07:53:15.920249Z	3
362	Airly_6013	49.1	2021-06-24T07:53:26.383485Z	3
452	Airly_7394	40.4	2022-01-03T11:03:20.048606Z	3

Supplementary Table 2: Studies on spatial correlation of $PM_{2.5}$ in urban environments.

Study	Location	Methodology	Conclusions
Burton, Suh and Koutrakis 51	Philadelphia, USA	Measured $PM_{2.5}$ at eight sites ranging from 0.6 to 28.8 km from the city center, summer 1992 and 1993	Pearson Correlation Coefficients between sites were $0.70 < r < 0.96$ for $PM_{2.5}$.
Wilson and Suh 52	St. Louis, USA	Measured $PM_{2.5}$ at four sites	High site-to-site correlations ($0.92 < r < 0.96$) for 24-h PM2.5 concentrations at the intraurban scale were correlated to population density.
DeGaetano and Doherty 53	New York City, USA	Measured $PM_{2.5}$ at a high-density network of 20 residential, commercial, and industrial stations between 2000 and 2002 — Instrument: Apered element oscillating microbalance (TEOM).	Slight spatial variation in concentration across the city. All but one of the sites in lower Manhattan had site-to-site correlations greater than 0.85.
Suh, Nishioka, Allen, Koutrakis and Burton 54	Washington, DC metropolitan area, USA	At six sites, in the summer of 1994	Correlations were high and significant for $PM_{2.5}$ ($0.69 < r < 0.98$)
Buzorius, Hämeri, Pekkanen and Kulmala 55	Helsinki, Finland	Four sites, from November 1st, 1996 until May 1st, 1997	In general, the number concentration time series measured in different places show a high correlation ($r > 0.7$).
Ye, Ji, Yang, Yao, Chan, Cadle, Chan and Mulawa 56	Shanghai, China	Two sites, Tongji University and Hainan Road, Starting in March 1999 and was conducted for one year	Average concentrations over the period at the two sites were 67.6 and 64.6 $\mu g\ m^{-3}$, with a high correlation value between sites ($r > 0.94$)

SI.1 uEMEP air quality model

The EMEP MSC-W model 57, created by the Meteorological Synthesizing Centre – West (MSC-W) of EMEP (the European Monitoring and Evaluation Program), is a chemistry transport model that operates within the framework of the United Nations Convention on Long-range Transboundary Air Pollution (LRTAP).

The uEMEP (urban EMEP) model employs an extension of the EMEP MSC-W chemical transport model to capture near-street scale dynamics, as described by Denby, Gauss, Wind, Mu, Grøtting Wærsted, Fagerli, Valdebenito and Klein 58. Within uEMEP, a combination of the classical Gaussian plume model and the physical parameterizations from the EMEP MSC-W model's emission data is utilized for downscaling. An illustrative application of the uEMEP approach is the Norwegian air quality forecasting and assessment system, which models the entire country at a resolution ranging from 250 to 50 m at hourly resolution. Comprehensive monitoring data from various locations, including traffic sites, are employed to validate the model. Validation outcomes reveal satisfactory results for NO_2 and moderate outcomes for $PM_{2.5}$ and PM_{10} 58. The authors define the validation outcomes for air pollutants NO_2, PM_{10}, and $PM_{2.5}$ based on the results for the year 2017. During this period, there were 72 operational air quality stations in Norway; however, not all stations measured all components. The total number of stations available for NO_2 and PM measurements with more than 75% coverage ranged between 34 and 45.

For NO_2, the spatial correlation is relatively high, r = 0.81 for uEMEP with slight negative Bias (Fractional Bias — FB of -5.9 %). The temporal variation over the year is also well represented when averaged over all stations (r = 0.79). For PM_{10}, the spatial correlation (r) between the model (uEMEP) and observations is relatively low at 0.29, with a negative Fractional Bias (FB) of -9.2%. The temporal variation over the year is well represented when averaged over all stations r = 0.61. Still, the model has a negative Bias of 4 $\mu g\, m^{-3}$ over the summer period. For $PM_{2.5}$, the spatial correlation (r) between the model (uEMEP) and observations is relatively good at 0.49, with a little negative Bias (FB = -10.5%). The temporal variation over the year is well represented when averaged over all stations (r = 0.67). Still, the model has a negative Bias of 2 $\mu g\, m^{-3}$ over much of the summer period.

RWC emissions in Norway and input data for the EMEP model are based on processing high-resolution datasets, including dwelling number and type, wood and other energy source

consumption, available heating technology, location of chimneys, and meteorology [11]. Residential emissions have been extensively validated for Norwegian cities by comparing modeling results with observations [11,59]. The model and/or its underlying input data do not fully capture the variations of air pollution at the high spatial resolution, and the assimilation of sensor data into the model can provide better estimates of the spatiotemporal variation of air pollution at regional scales.

SI.2 Airly sensor systems

The Airly sensor kit measures temperature and RH with a DHT22 temperature/RH sensor. The Airly unit delivers hourly averaged data; however, the original sampling rate is one measurement per five minutes. The data is communicated using the Global System for Mobile Communications (GSM) and transferred to a Norwegian Institute for Air Research (NILU) sensor data platform for further analysis (https://nordicpathlive.nilu.no/, accessed in Nov 2022). Additionally, the sensor system is already assembled/mounted and must only be connected to the power supply. It has a weatherproof casing with dimensions of 74 × 77 × 83.5 mm and a weight of 440g. According to the manufacturer, the sensor system can measure PM with a declared accuracy of ±10 µg m^{-3} in the three fractions; it operates in the temperature range between -10 and +60 °C and RH range of 0 - 100%. Airly (optical) PM sensors measure the micro-particle concentration (aerodynamic diameters of 0.3 - 10 µm) through the light-scattering principles; the sensor's effective detection range is 0 to 500 µg m^{-3}.

Among the early and limited studies evaluating the performance of Airly PM LCSs, Vogt, Schneider, Castell and Hamer 25 analyzed the performance of three Airly sensors against reference instrument FIDAS 200 (Palas GmbH, Germany) at *Kirkeveien* air quality station in Oslo (2020-09-09 until 2020-10-19); the results showed a post-calibration MB and RMSE between 1.37 and 7.89 µg m^{-3} and 4.39 and 10.9 µg m^{-3} for $PM_{2.5}$, respectively. However, the Airly PM sensor relatively showed a post-calibration poor performance measuring PM_{10} (MB = 3.19 - 9.71 µg m^{-3}, and RMSE = 5.26 - 12.15 µg m^{-3}). This behavior is typical for other PM sensor systems using light scattering methods, and recent literature shows that PM LCSs have better performance for $PM_{2.5}$ than PM_{10} 37, 60. However, all the evaluation metrics in Vogt, Schneider, Castell and Hamer 25 analysis, such as MB or RMSE, are based on the calibrated data, not the raw output of the sensor.

Correspondingly, the results of applying the sensor-specific multilinear regression models by Vogt, Schneider, Castell and Hamer 25 to three Airly sensor systems against reference gravimetric method in the field co-location at the *Kirkeveien* also showed that sensors overestimate the 24-h averages of $PM_{2.5}$ and division by scale factors of 2.08, 1.87, and 2.46 is required to correct the data (Theil slope with an intercept of zero). For comparison, we found a slope scale factor of 1.88, with intercept equal to zero. The evaluation of the dependency of the sensors on RH against reference-equivalent optical instrument FIDAS 200 (Palas GmbH, Germany) showed a

change in Bias for RH exceeding 70%, with the most considerable Bias being 5 - 10 μg m^{-3} when the RH reaches a value of 100%.

During a co-location campaign between 2021-01-22 and 2021-02-10, three Airly PM LCSs were installed by Hofman, Peters, Stroobants, Elst, Baeyens, Van Laer, Spruyt, Van Essche, Delbare and Roels 31 on the roof of a regulatory air quality station in Antwerp, Belgium equipped with a FIDAS 200, Palas reference-grade instrument. They found slope calibration factors (Sensor = scale factor × Ref) of 1.91, 1.63, and 1.90 for the three sensors' $PM_{2.5}$ measurements (sensor-to-sensor *r*s between the three Airly sensors were between 0.986 and 0.989). The pre-calibration *r*s, MAEs, and RMSEs between the three Airly sensors and the reference measurements were 0.89 - 0.92, 9.63 - 13.58 μg m^{-3}, and 11.72 - 16.45 μg m^{-3}, respectively. However, they ran another calibration campaign in late Spring and early summer (2021-05-18 until 2021-06-08) and found that sensors underestimated the $PM_{2.5}$ and the slope scale factors were respectively 0.75, 0.70, and 0.73 (sensor-to-sensor *r*s between the three Airly sensors were between 0.921 and 0.956. We have access to the data of a co-location campaign at *Bjørndalssletta* reference station equipped with Grimm EDM180 optical dust monitor in Kristiansand during the summer of 2022 (2022-07-25 until 2022-08-13), represented in Supplementary Figure 20a. We also analyzed the results of the co-location at *Haga* station in Gothenburg during the same period in the summer of 2022 (Supplementary Figure 20b). We see similar results here, i.e., overall better performance and underestimation of $PM_{2.5}$ during the summer. Hofman, Peters, Stroobants, Elst, Baeyens, Van Laer, Spruyt, Van Essche, Delbare and Roels 31 attribute this to less RH during the summer.

SI.3 Small Filter Devices — Kleinfiltergerät LVS3 and MVS6

The KFG is an integrated, gravimetric method intended to provide measurements of either fine PM concentration, PM_1 or $PM_{2.5}$ (according to CEN EN 14907 standard) or larger particles mass concentrations, PM_{10} (CEN EN 12341 standard) over a 24-hour sampling interval. An ambient air sample is collected by an electrically powered sampler operating at a constant volumetric flow rate. Sample air is drawn from the atmosphere at 38.33 L min^{-1} (2.3 m^3 h^{-1}) through an inlet designed to reject insects and atmospheric precipitation and to be insensitive to wind speed and direction. This sample filter is conditioned and manually weighed before and after sample collection to determine the increase in mass. The net mass gain is divided by the measured sample volume to determine the mass concentration of either $PM_{2.5}$ or PM_{10}. Further technical details on the KFG device are available as Supplementary Information.

Leberstraße 63 | +49 (0) 30 78 95 50 11 | info@leckel.de | **SVEN LECKEL**
10829 Berlin/Germany | +49 (0) 30 78 95 50 12 | www.leckel.de | Ingenieurbüro GmbH

SMALL FILTER DEVICE (Kleinfiltergerät) LVS3

SMALL FILTER DEVICE (Kleinfiltergerät) MVS6

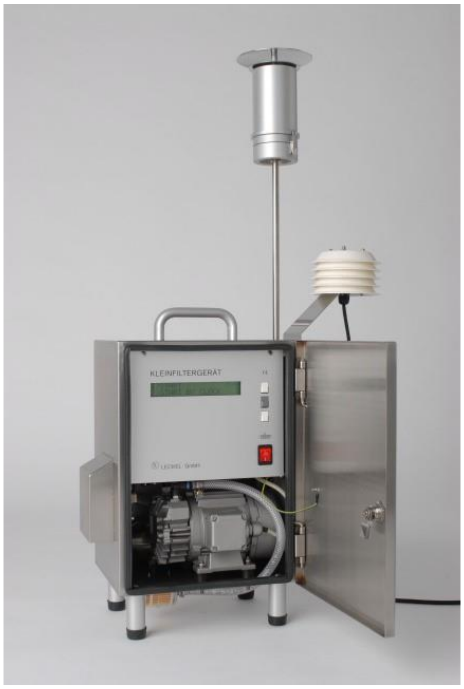

Features

Rugged, light-weight construction for outdoor use (stainless steel), small set-up area

The device will be automatically heated (Winter operation) and ventilated

Controlling of **operating-m³/h** (ambient air conditions) and standard-**m³/h** (0 °C or 20 °C, 760 mm Hg) by an orifice plate

Impactor inlets with exchangeable jets (8 pieces) for **PM10 – PM4,0 – PM2,5 – PM1,0**

Inlets for TSP, PU foam (with and without ozone denuder) and bioaerosols

Easy and self-explanatory 3-key menu-guided operation

Data storage on **memory stick**

• Bavarian-Hessian protocol

Pre-selectable activation, sampling duration and sampling intervals

Protection of stored data against power failure, real-time clock

External set-up of inlets

Use of filters with diameters of **47 mm** and **50 mm**

REFERENCE SAMPLER

according to

VDI 2463 Parts 7 und 8
Total dust measurement by using the
STANDARD INLET

VDI 2465 Part 1
Soot (EC) measurement by using the
PM10 INLET

CEN EN 12341
PM10 measurement

PM2,5 STANDARD INLET (IMPAKTOR)
according to CEN EN 14907

Description

The Small Filter Devices (Kleinfiltergeräte) **LVS3** and **MVS6** are designed for outdoor use at very high as well as very low temperatures. The devices can also be used indoors.

The flow rates of the samplers are controlled in compliance with basic physical principles by means of a temperature- and pressure-compensated orifice plate according to Bernoulli's law and by conversion into operating-m³/h resp. standard-m³/h according to BoyleMariotte`s law. The sampled air volume is displayed in operating-m³ and standard-m³ with a sensitivity of 0,01 m³ on the digital display. In case of a pressure drop across the filter of more than 300 mbar the device will automatically shut down.

All relevant data are shown on the display and can be stored on a memory stick. In case of a power failure, all data stored in the micro controller and in the system's memory will be safe for several years thanks to a built-in high-capacity battery.

The device's housing consists of stainless-steel sheet metal with a lockable door. The device's solid construction guarantees a high availability.

Because of their low noise emission level, the Small Filter Devices can be used in urban areas at any time of the day and indoors as well. The sampling head can also be set up externally, e.g.,. directly at kerbsides or in living rooms by using a hose connection to the instrument placed in a greater distance.

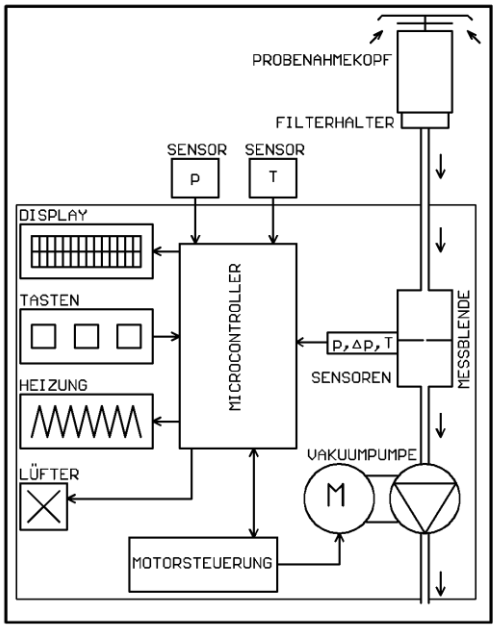

BLOCKSCHALTBILD LVS3 / MVS6

Subject to alterations Ed. 07/11

Model Variations

LVS3
This model can be operated with controlled flow rates between 1,0 and 2,3 m³/h. In the uncontrolled mode (UMODE), the device is identical with regard to its function to the type GS 050/3 described in the guideline VDI 2463 Part 7.

MVS6
This model can be operated with controlled flow rates between 2,3 and 3,5 m³/h. Its design is identical with the model LVS3. The controlled flow rate of 2,7 m³/h meets the requirements of VDI 2463 parts 7 and 8.

Inlets
For ambient air, indoor and workplace measurements

- Measurement of PM10 (EN12341) and PM2,5 (EN14907)
- Measurement of PM4,0 and PM1,0
- Measurement of TSP (VDI 2463-8)
- Measurement of heavy metals (VDI 2267 and EN14902))
- Measurement of PCBs (VDI 2464-1)
- Measurement of soot (EC/OC) (VDI 2465)
- Measurement of dioxins and furans (VDI 3498-2)
- Measurement of bioaerosols (VDI 4252-2)
- Measurement of PAHs, PCDD, PCDF, PCB and house dust (VDI 4300 and ISO 16000-13)
- Measurement of lindane/PCP, house dust etc. (VDI 4301)
- Measurement of BaP (Scrubber/EN 15 549)

The dust collected on the filters can also be analysed on ions (sulphate, nitrate etc.) as well as radioactivity.

Technical Data
Flow rate

LVS3 uncontrolled approx. 3,2 m³/h
controlled 1,0-1,6-2,0-2,3 m³/h
and standard-m³/h
Deviation from the set point: < 2%

MVS6 uncontrolled approx. 5 m³/h
Controlled 2,3-2,7-3,0-3,5 m³/h
and standard-m³/h
Deviation from the set point: < 2%

Sampling time minimum
1h = maximum 999 h

Power supply
230 V, 50/60 Hz

Consumption
LVS3 approx. 250 VA – **MVS6** approx. 300 VA

Filter diameter
47 mm and 50 mm

Diameter of loaded filter surface
approx. 40 mm

Dimensions
Width 310 mm – Height 480 mm – Depth 250 mm

Weight
LVS3 approx. 22 kg **– MVS6** approx. 23 kg

Noise level according to DIN 2058
<< 35 dBA

SI.4 Optimal Interpolation

OI and geostatistical techniques, such as universal kriging or kriging with external drift 61, are closely linked. Following the nomenclature used in Kalnay 29, we calculate the analysis vector $\mathbf{x}_a$ as:

$$\mathbf{x}_a = \mathbf{x}_b + \mathbf{W}[\mathbf{y}_\mathrm{o} - H(\mathbf{x}_b)]$$

where $\mathbf{x}_b$ is the background field vector from a model, $\mathbf{W}$ is a matrix of weights, $\mathbf{y}_o$ is the set of observations obtained from the LCS network, and H is the observation operator that translates the background values into observation space (often using simple bilinear interpolation if the units of the background field and the observations are the same). The weight matrix $\mathbf{W}$ is calculated as:

$$\mathbf{W} = \mathbf{BH}^T(\mathbf{R} + \mathbf{HBH}^T)^{-1}$$

where **B** is the background error covariance matrix, the matrix **H** is the linear tangent perturbation of H, and **R** is the matrix of observation error covariances (which is diagonal if the observations errors at different locations are assumed to be un*r*elated).

The analysis error covariance $\mathbf{P}_a$ can then be calculated as

$$\mathbf{P}_a = (\mathbf{I} - \mathbf{WH})\mathbf{B}$$

where **I** is the identity matrix.

One of the most complex challenges in applying the OI in practice, particularly for urban-scale applications, as we do here, is to design the background error covariance matrix B. We apply a method that uses spatial auto-correlation within the model field overlaid with a distance decay function. This provides a “spatial representativity footprint” for each observation site, determining how the signal observed at the measurement sites should spread in space. As a model, we used the operational forecasts of the uEMEP model 58, 62 provided by the Norwegian Meteorological Institute.
